# Task-irrelevant decorative pictures increase cognitive load during text processing but have no effects on learning or working memory performance: an EEG and eye-tracking study

**DOI:** 10.1007/s00426-024-01939-8

**Published:** 2024-03-19

**Authors:** Christian Scharinger

**Affiliations:** Leibniz-Institut für Wissensmedien Tübingen, Schleichstr. 6, 72076 Tübingen, Germany

## Abstract

Decorative pictures (DP) are often used in multimedia task materials and are commonly considered so-called seductive details as they are commonly not task-relevant. Typically, DP result in mixed effects on behavioral performance measures. The current study focused on the effects of DP on the cognitive load during text reading and working memory task performance. The theta and alpha frequency band power of the electroencephalogram (EEG) and pupil dilation served as proxies of cognitive load. The number of fixations, mean fixation durations, and the number of transitions served as proxies of the attentional focus. For both, text reading and n-back working memory tasks, the presence and congruency of DP were manipulated in four task conditions. DP did neither affect behavioral performance nor subjective ratings of emotional–motivational factors. However, in both tasks, DP increased the cognitive load as revealed by the EEG alpha frequency band power and (at least to some extent) by subjective effort ratings. Notably, the EEG alpha frequency band power was a quite reliable and sensitive proxy of cognitive load. Analyzing the EEG data stimulus-locked and fixation-related, the EEG alpha frequency band power revealed a difference in global and local cognitive load. In sum, the current study underlines the feasibility and use of EEG for multimedia research, especially when combined with eye-tracking.

## Introduction

Decorative pictures (DP) accompanying texts are widely used not only in the digital informational environments of the World Wide Web (e.g., the digital encyclopedia wikipedia.org) but also in traditional knowledge media like printed science textbooks for school students (e.g., textbooks of biology; Wiley et al., 2017). DP are commonly only moderately content-related, not necessarily needed for understanding the learning content (i.e., not task-relevant) and are, therefore, often considered so-called seductive details (Rey, 2012; Sundararajan & Adesope, 2020). Intuitively plausible, DP may function as an eye-catcher, to increase the learners’ interest in the content domain and the topic and to increase the general aesthetical pleasantness of the task materials (Carney & Levin, 2002; Rey, 2012; Takahashi, 1995). Often used as DP are photographs of (famous) humans or (pleasant) landscapes, that is, motifs that are typically perceived as interesting and aesthetically pleasant by humans (Vessel & Rubin, 2010).

However, concerning text comprehension and learning outcomes, research on DP as seductive details revealed mixed results, ranging from studies reporting beneficial effects (Schneider et al., 2016, 2018a, 2018b) to no effects (Chang & Choi, 2014; Lenzner et al., 2013; Lindner, 2020; Park & Lim, 2007) and even detrimental effects (Bartsch & Cobern, 2003; Chang & Choi, 2014; Harp & Mayer, 1997; Magner et al., 2014; Rey, 2014; Sanchez & Wiley, 2006). While beneficial effects of DP on learning are mainly attributed to emotional–motivational factors (see *emotional design hypothesis*; e.g., Brom et al., 2018; Heidig et al., 2015), cognitive factors such as an increased load on attention control and working memory (WM), the disruption of building a coherent mental model, and the interference between textual and pictorial schemata have been discussed as potentially underlying the detrimental effects of DP on learning (Rey, 2012; Sundararajan & Adesope, 2020). All these potential explanations share the underlying or directly stated assumption of increased cognitive load for seductive details like DP. Despite the central role of cognitive load in the seductive details effect, studies to date reported predominantly subjective measures thereof (i.e., assessed via rating scales; Schneider et al., 2018a, 2018b; Sundararajan & Adesope, 2020). A major disadvantage of subjective rating scales to assess cognitive load is that they only provide insights into participants’ post hoc evaluation of their subjectively experienced cognitive load in a previously finished task. Apart from subjective biases, it therefore remains elusive whether subjective ratings measure an average of the cognitive load experienced during the previous task or a particular peak load that participants might remember best (Ayres, 2017; Schmeck et al., 2015). Physiological process measures such as the electroencephalogram (EEG) and more specifically the EEG theta (4–6 Hz) and alpha (8–13 Hz) frequency band power can overcome such constraints by allowing to assess the actual cognitive load during a task objectively (Antonenko et al., 2010; Gevins et al., 1997; Pesonen et al., 2007; Scharinger et al., 2015a, 2015b).

Using EEG frequency band power measures and pupil dilation, the current study specifically focused on assessing the effects of DP on cognitive load during text processing and WM task performance. Notably, the EEG data for text processing were not only analyzed in the traditionally overall, stimulus-locked manner but also fixation-related, that is, specifically for fixations on the DP. The combination of EEG *and* eye-tracking data has been studied for about a decade, yet predominantly focusing on fixation-related potentials (e.g., Baccino, 2011; Dimigen et al., 2011; Ossandon et al., 2010). Yet, focusing on EEG frequency band power fixation-related as a measure of cognitive load has not been reported before in studies on seductive details like DP. In addition, in the current study, pupil dilation was analyzed as an easily acquired measure (compared to EEG) for the cognitive load (Cabestrero et al., 2009; Kahneman & Beatty, 1966; Laeng et al., 2012; Mathôt, 2018; van der Wel & van Steenbergen, 2018). Yet, pupil dilation is prone to luminance confounds (Van Gerven et al., 2004), which might specifically be problematic when examining multimedia task materials (i.e., combinations of text and pictures). As a minor research question, the current study was interested in whether pupil dilation might provide reasonable cognitive load estimations even in the context of more complex task materials. Finally, eye-tracking served as a measure of attentional focus (Just & Carpenter, 1980; Rayner, 2009) to assess the effects of DP on attention control (i.e., distractive effects of DP).

## Physiological proxies of attention and cognitive load

Eye-tracking is a well-established and widely used methodology in research on reading (e.g., Rayner, 1998, 2009) and multimedia learning (e.g., Alemdag & Cagiltay, 2018; Hyönä, 2010; Lai et al., 2013; Scheiter & Eitel, 2017; van Gog & Scheiter, 2010). Among others, eye-tracking allows to measure the number and duration of eye fixations on specific areas of interest (AOIs) on the screen (e.g., text or pictures). It is generally assumed that those visual elements on the screen that are fixated at are in the focus of attention and are processed (i.e., the so-called eye-mind assumption; Just & Carpenter, 1976, 1980; Rayner, 2009). Thus, eye fixations may serve as an indicator of attentional focus, with the number of fixations and fixation duration indicating the interestingness and informativeness of certain AOIs and the depth of processing (Rayner, 2009). Especially, mean fixation durations might serve as a proxy for indexing cognitive load (i.e., longer fixation durations might indicate increased cognitive load; Rayner, 2009). The number of transitions between text and picture (i.e., the number of direct switches between fixations on the text and fixations on the picture) might be highly informative regarding the integration of textual and pictorial information (i.e., processes of constructing a coherent mental model during reading). Higher numbers of transitions indicate stronger efforts of integrating textual and pictorial information (Mason et al., 2013a, 2013b; Schüler, 2017).

Pupil dilation (i.e., the size of the eyes’ pupils) can serve as a proxy of cognitive load (Cabestrero et al., 2009; Kahneman & Beatty, 1966; Laeng et al., 2012; Mathôt, 2018; van der Wel & van Steenbergen, 2018). Typically, the pupil dilates for increasing cognitive load. Yet, differences in luminance on the screen can confound pupil dilation data (Van Gerven et al., 2004), which might especially be problematic when examining multimedia task materials (i.e., combinations of text and pictures). Furthermore, for technical reasons, the shape of the pupil assessed by the eye tracker differs depending on the gaze position, consequently affecting the size estimation of the pupil (see the *pupil foreshortening error*, e.g., Hayes & Petrov, 2016; Mahanama et al., 2022; Petersch & Dierkes, 2022). This has to be kept in mind as a potential additional confounding factor of the pupil dilation data in free-viewing situations like the reading tasks of the current study. Nevertheless, pupil dilation might be a very interesting proxy of cognitive load for multimedia materials. This is because pupil dilation can be easily acquired alongside eye-tracking data.

The EEG alpha and theta frequency band power might overcome the constraints of pupil dilation as a proxy of cognitive load. Typically, for increased cognitive load, an increase of frontal midline EEG theta power (i.e., typically at electrode Fz) and a decrease of parietal EEG alpha power (typically at electrode Pz) have been reported in a variety of tasks, not only rather basic, highly controlled WM tasks like the n-back task (e.g., Brouwer et al., 2014; Fairclough & Ewing, 2017; Gevins et al., 1997; Palomäki et al., 2012; Scharinger et al., 2015a, 2015b) but also for more complex, instructional task materials. These seminal studies examined, for example, the effects of leads (i.e., short previews) on hypertext reading (Antonenko & Niederhauser, 2010), hyperlink-selection processes (Scharinger et al., 2015a, 2015b), the spatial contiguity effect in multimedia learning (Makransky et al., 2019), the integration of written and pictorial information (Scharinger et al., 2020), or learning in dyads (Mercier et al., 2021). Yet, to date, EEG frequency band power has not been used to study the effects of DP on cognitive load.

## Effects of pictures in learning materials on cognitive load

Pictures alongside text are generally assumed to be processed by the reader and, if possible, to be integrated into a coherent mental model (Kintsch, 1988; Mayer, 2014a; Schnotz, 2014; Schüler, 2017; Schüler et al., 2015). The processing of the pictorial and textual information and the integration with prior knowledge, that is, the construction of a coherent mental model, are thought to take place in WM (cf. the *Cognitive Theory of Multimedia Learning*, Mayer, 2014a) or the *Integrated Model of Text and Picture Comprehension*, Schnotz, 2014), thus requiring corresponding resources (Sanchez & Wiley, 2006). As WM is of limited capacity (Baddeley, 2012; Cowan, 2010), DP consume WM resources that otherwise would be available for processing the core learning content. Thus, from a cognitive perspective, adding DP should result in detrimental effects on learning and task performance.

Important to note that DP might be differentiated from instructional pictures. The latter are pictures closely related to the learning content and helpful (or even necessary) for understanding the textual information. The effects of instructional pictures have been intensively studied in multimedia research (e.g., Alemdag & Cagiltay, 2018). Instructional pictures are, if adequately designed, thought to substantially contribute to understanding the learning content (cf. *multimedia principle*; Mayer, 2002, 2014b; Mayer & Moreno, 2003) and hence are mostly considered beneficial for learning. In contrast, DP are considered detrimental to learning because of being redundant or content-unrelated. Yet, a clear-cut differentiation between DP and instructional pictures might not always be possible (e.g., instructional pictures might also be decorative; Heidig et al., 2015; Um et al., 2012). Thus, pictures’ decorative and instructional character might not be considered exclusive but rather as two related, orthogonal dimensions on which pictures can be situated (Lenzner et al., 2013).

Depending on the semantic content-relatedness of pictures and text, the integration of textual and pictorial information and the construction of a coherent mental model might be facilitated or hampered (Scharinger et al., 2020; Schüler et al., 2015). With DP per definition being rather loosely content-related, the construction of a coherent mental model might be specifically hampered, resulting in increased cognitive load while reading with DP. In a series of three consecutive experiments, Schneider and colleagues manipulated in a two-by-two design with the control group (i.e., a text-only condition), the valence (negative versus positive valence) of DP, and the content-relatedness (weakly versus strongly content-related pictures; Schneider et al., 2018a, 2018b). Overall, task materials with positive or strongly content-related DP resulted in better learning outcomes as compared to negative or weakly content-related DP (Schneider et al., 2018a, 2018b). In two of the experiments (Experiment 2 and 3), the authors also assessed subjective ratings of cognitive load, additionally differentiating between intrinsic and extraneous cognitive load (Sweller et al., 1998). Items to assess intrinsic cognitive load focused on aspects of the learning content, whereas items to assess extraneous cognitive load focused on aspects of the task instruction. DP of negative valence resulted in increased cognitive load (both intrinsic and extraneous) compared to DP with positive valance, irrespective of content-relatedness. In contrast, only intrinsic cognitive load was affected by content-relation with weakly content-related DP resulting in higher subjectively perceived intrinsic cognitive load. However, as the study by Schneider and colleagues only used subjective measures of cognitive load, it remains an open question whether the weakly content-related DP indeed increased cognitive load (i.e., objectively measured) or whether they only influenced the subjective ratings (i.e., the subjectively *perceived* load). Thus, building on these behavioral results, the current study aimed at assessing the effects of DP with varying content-relatedness on cognitive load, objectively measured. Note that physiological measures such as the EEG alpha and theta frequency band power only provide a global proxy of cognitive load. To my knowledge, any differentiation in intrinsic or extraneous aspects of cognitive load based on these measures is not possible. Therefore, in the current study, intrinsic and extraneous cognitive load were not differentiated. Furthermore, it is important to keep in mind that the EEG measures and pupil dilation are not specific to fluctuations in cognitive load only (e.g., Klimesch, 1999; also, see limitation section below). Yet, typically they do react to fluctuations in cognitive load. For reasons of readability, in the current manuscript EEG theta and alpha frequency band power and pupil dilation are therefore termed proxies of cognitive load.

Apart from hampering the construction of a coherent mental model, there are at least two other reasons why DP might increase cognitive load. First, the cognitive load might be increased simply because of the additional information that must be processed when DP are present (i.e., DP might be considered extraneous cognitive load; Sweller et al., 1998). Second, the cognitive load might be increased because of DP distracting the readers’ attentional focus away from the text (Rey, 2012). Attention-related, executive control functions such as inhibition and shifting (Miyake et al., 2000) might be demanded as readers might shift their attentional focus between text reading and picture viewing, or readers might try to inhibit the DP (also see recent assumptions on the role of self-regulation for reading performance; Daucourt et al., 2018; Nejadihassan & Arabmofrad, 2016). The assumption that DP might be inhibited has been supported by eye-tracking studies indicating that DP commonly only got a bit of (initial) attention and then that were mainly ignored (Chang & Choi, 2014; Lenzner et al., 2013; Rey, 2014). The combination of EEG and eye-tracking, as done in the current study, thus seems promising to assess the effects of DP on the attentional focus (i.e., by eye-tracking) and cognitive load (i.e., by the EEG).

## The present study

The present study examined the effects of DP on cognitive load during text processing (i.e., learning tasks) and WM processing (i.e., n-back WM tasks). The EEG theta and alpha frequency band power and pupil dilation served as proxies of cognitive load. It was expected that the increased cognitive load for DP present would result in an increased EEG theta frequency band power at frontal electrode Fz, a decreased EEG alpha frequency band power at parietal electrode Pz, and an increased pupil dilation (Gevins & Smith, 2000; Kahneman & Beatty, 1966; Palomäki et al., 2012; Scharinger et al., 2015a, 2015b). For that, in a complete within-subjects design, the presence of DP alongside text in learning tasks and n-back stimuli in WM tasks, respectively, was manipulated.

The manipulation of DP not only in text reading tasks but also in n-back WM tasks was done for two reasons. First, the n-back task is a well-established paradigm in neurophysiological research to study cognitive load (Brouwer et al., 2012; Gevins & Smith, 2000; Owen et al., 2005; Palomäki et al., 2012; Pesonen et al., 2007; Scharinger et al., 2015a, 2015b). Thus, the n-back task was sought to serve as a well-controlled testbed to assess the effects of cognitive load on the (neuro-) physiological measures (and to avoid potential confounds; see Gerjets et al., 2014). Second, as described above, WM and its limited capacity are seen as the fundamental construct in theories on text–picture processing and multimedia learning (Mayer, 2014a; Schnotz, 2014). Furthermore, attentional executive control functions such as inhibition, shifting, and updating (Miyake et al., 2000) are closely linked to WM (Baddeley, 1996). Thus, conceptually, WM might play a role not only in the processing and integration of DP but also in the inhibition of distracting information and shifting between the processing of pictorial information and the primary task. Therefore, the current study was not only interested in whether DP would affect cognitive load but also in whether DP would directly affect WM processing, that is, resulting in detrimental effects on WM performance (i.e., decreased n-back accuracies and increased reaction times for DP present as compared to DP absent). To my knowledge, the current study is the first to add DP spatially aside from the n-back stimuli (Ribeiro et al., 2019). Furthermore, it used convenient DP, that is, pictures that we experience in our everyday media life and not pictures of artificially high valence and arousal levels like those from the IAPS database (Lang et al., 2008). Finally, the n-back task allowed to exploratorily study the potential effects of DP under low (0-back) and high (2-back) WM load.

Depending on the congruency between pictorial and textual information, the DP might differently affect learning outcomes and cognitive load (Schneider et al., 2018a, 2018b). Semantically strongly related DP might benefit learning more than weakly related DP (Schneider et al., 2018a, 2018b). To examine these potential effects, the semantic relatedness of the DP was additionally manipulated in the current study resulting in four task conditions concerning DP. As learning task materials served four texts providing factual knowledge on habits and habitats of animals, one animal per text (i.e., polar bear, arctic fox, lion, zebra). The *congruent condition* (CC) consisted of DP (realistic photographs) depicting landscapes or animals content-congruent to the corresponding text. Important to note that the DP were semantically related to the text, but not needed for text comprehension. Comparably, words (nouns) were used as n-back stimuli that were semantically related to the DP. The *incongruent condition* (IC) consisted of DP (realistic photographs) depicting landscapes or animals content-incongruent with the corresponding text or n-back stimuli (e.g., DP of polar regions combined with the text about lions). The *distractor condition* (DC) consisted of semantically unrelated, grayscale images depicting optical illusions (e.g., drawings evoking illusory movements) presented alongside the texts and n-back stimuli, respectively. The optical illusions were thought to be visually highly interesting (Stevanov et al., 2012a, 2012b), thus attention-capturing. The DC was added to the current study for two reasons. First, in this condition, the DP were grayscale only, depicting abstract forms. Concerning pupil dilation potential luminance confounds might be less severe in this condition as compared to the CC and IC using realistic color photographs. Second, in the DC, the DP were completely unrelated to the text (not simply showing other animals or habitats as in the IC). Therefore, this condition was considered to be maximally distracting. Finally, the *no-picture condition* (NC) consisted of pure texts or n-back stimuli without any DP.

I expected all task conditions with DP present to result in increased cognitive load (i.e., increased EEG theta power at electrode Fz, decreased EEG alpha power at electrode Pz, and increased pupil dilation) compared to the task condition without DP. Furthermore, task conditions IC and DC with semantically unrelated DP might result in a higher cognitive load than task condition CC with semantically related DP. This is because, only in the CC, textual and pictorial information being congruent, the construction of a coherent mental model might be easily possible. In contrast, the IC and DC might impede the construction of a coherent mental model. In addition, because of the conflicting information, the DP of the IC and DC might be more attention-capturing (i.e., more distractive) than those of the CC in both, the learning and the n-back WM tasks, consequently leading to increased cognitive load.

Especially promising in research on instructional (multimedia) material is the combined analysis of EEG and eye-tracking data, that is, using the eye-tracking data to analyze the EEG frequency band power in relation to fixations on certain AOIs (Baccino, 2011; Dimigen et al., 2011; Ossandon et al., 2010; Scharinger, 2018; Scharinger et al., 2020). In classical, stimulus-locked EEG data analyses, EEG frequency band power is calculated for data epochs (i.e., time windows) linked to the onset of the stimuli (e.g., the start of the entire text or the beginning of each paragraph or otherwise defined time window in multimedia text materials). For multimedia materials, a stimulus-locked EEG analysis might provide the overall cognitive load of the task. However, fluctuations in cognitive load during learning (Paas et al., 2003) might be averaged out using this data selection procedure. Instead, the fixation-related EEG data analysis allow assessing the cognitive load more fine-grained when certain elements on the screen (i.e., a defined AOI like the DP) are fixated at. This is because, eye-fixations are thought to signal the processing of the fixated visual content (Hyönä, 2010; Just & Carpenter, 1980; Rayner, 2009). Thus, this methodology might allow closely tapping into specific cognitive load associated with specific AOIs. Therefore, in the current study, I exploratorily analyzed the EEG theta and alpha frequency band power and pupil dilation fixation-related, that is, for fixations on the DP (i.e., the conditions CC, IC and DC). As it was an exploratory analysis, I had no strong a priori expectations regarding outcomes.

Also without a strong priory expectation, the eye-tracking data was analyzed as a proxy of the attentional focus (i.e., number of fixations on DP, mean fixation duration) and text–picture integration (i.e., the number of transitions; Mason, Pluchino, et al., 2013; Mason, Tornatora, et al., 2013). Finally, for getting a comprehensive picture, task performance (i.e., text reading times, n-back reaction times, n-back accuracies) and subjective task experience (e.g., subjectively perceived effort; NASA task load index, NTLX; Hart & Staveland, 1988) were assessed in the current study. Also exploratorily, to provide a thorough analysis of the different measures of cognitive load, the correlation between pupil dilation, EEG theta and alpha frequency band power and the subjective ratings of cognitive load was examined.

In sum, the current study examined the effects of DP on the cognitive load (both objectively and subjectively assessed) on learning outcomes, WM performance, and attentional focus. Using EEG in combination with eye-tracking and using a comparable manipulation of DP in text reading and n-back WM tasks extended previous studies on DP and the seductive details effect. In doing so, the current study was thought to methodologically and empirically advance research on DP in digital media, potentially leading to a more thorough understanding of the effects of DP on reading comprehension and WM performance.

## Method

### Participants

Thirty-two healthy subjects (university students, mean age = 23.38, *SD* = 2.81, 24 females, seven males, one n.s.) participated in the study for a payment of 10 € per hour. They were all German native speakers, right-handed as indicated by the Edinburgh Handedness Inventory (Oldfield, 1971), had normal or corrected-to-normal visual acuity, and reported no known visual or neurological disorders (e.g., color blindness or epilepsy). All participants had a school education of eight to nine years, completed with a high school graduation. The sample size was set based on the expectation of mid-sized effects for the physiological measures (partial eta square > 0.12), an alpha error probability of *α* = 0.05, and a statistical power of 0.95 for the present experimental design as calculated by G*Power (Faul et al., 2007).

The study has been approved by the local ethics committee and was conducted in accordance with the ethical standards laid down in the 1964 Declaration of Helsinki and its later amendments or comparable ethical standards. All participants gave their written informed consent before their inclusion in the study. Data collection took place during the COVID-19 pandemic in Europe in compliance with corresponding hygienic and health regulations (e.g., participants wearing medical masks). Only subjects with no academic background in biology or geography (i.e., the content domain of the learning tasks of the current study) were invited. Furthermore, participants’ prior knowledge and content interest were assessed using subjective ratings. Rating scales ranging from 0 (= no prior knowledge) to 100 (= expert knowledge) revealed a relatively low prior knowledge (*M* = 17.50, *SD* = 12.89). Participants’ general interest in biological topics (e.g., flora and fauna) was slightly below the mid of the corresponding rating scale (*M* = 3.79, *SD* = 1.07; 7-point Likert scales). Participants’ specific interest in the topics of the learning tasks (i.e., their interest in learning more about it) was slightly above the mid of the corresponding rating scale (*M* = 4.73, *SD* = 1.17; 7-point Likert scales).

## Materials

### Learning tasks

The learning materials consisted of four texts, each describing in Wikipedia-like writing style the body features, behavior, and habitat of a specific animal. Two described animals had cold, northern polar regions as habitats (arctic fox, polar bear), and two had warm, southern savannah regions as habitats (lion, zebra). The texts were based on German Wikipedia articles (Wikipedia.de) yet without hyperlinks. Texts were, on average, 956.75 (*SD* = 39.75) words long, separated into 20 single paragraphs, justified print, each paragraph containing, on average, 336.13 (*SD* = 50.13) letters (including spaces). The single paragraphs were positioned centrally on a 1920 × 1200 screen, with a width of the text area of 580 px (12.80° visual angle) and a height (depending on the number of letters per paragraph) ranging between 140 and 300 px (3.10° to 6.64° visual angle). The texts were written in black (font type: Arial, font size: 20 px) on a mid-gray (RGB: 145,145,133) background.

For each participant, each text was assigned to one out of four specific task conditions concerning DP. The assignment of the four texts to the four task conditions and the sequence of the task conditions were counterbalanced across participants. In the *congruent condition* (CC), 20 color photographs were presented aside from the paragraphs of the text depicting either the animal described in the text or the landscape of its habitat (4 DP landscape, 16 DP animal). In the *incongruent condition* (IC), each text paragraph was presented with a content-incongruent DP. Content incongruency was achieved by combining the texts about the northern polar animals with the DP of the southern savannah (4 DP landscape, 16 DP animal) and vice versa. Important to note that none of the DP was presented twice within the learning tasks for a specific participant. In the *distractor condition* (DC), each paragraph of the text was presented with a content-unrelated distractor picture. As distractor pictures served 20 grayscale pictures of optical illusions (e.g., a drawing evoking illusory movements). Finally, the text was presented without pictures in *the no-picture condition* (NC).

**Fig. 1:**
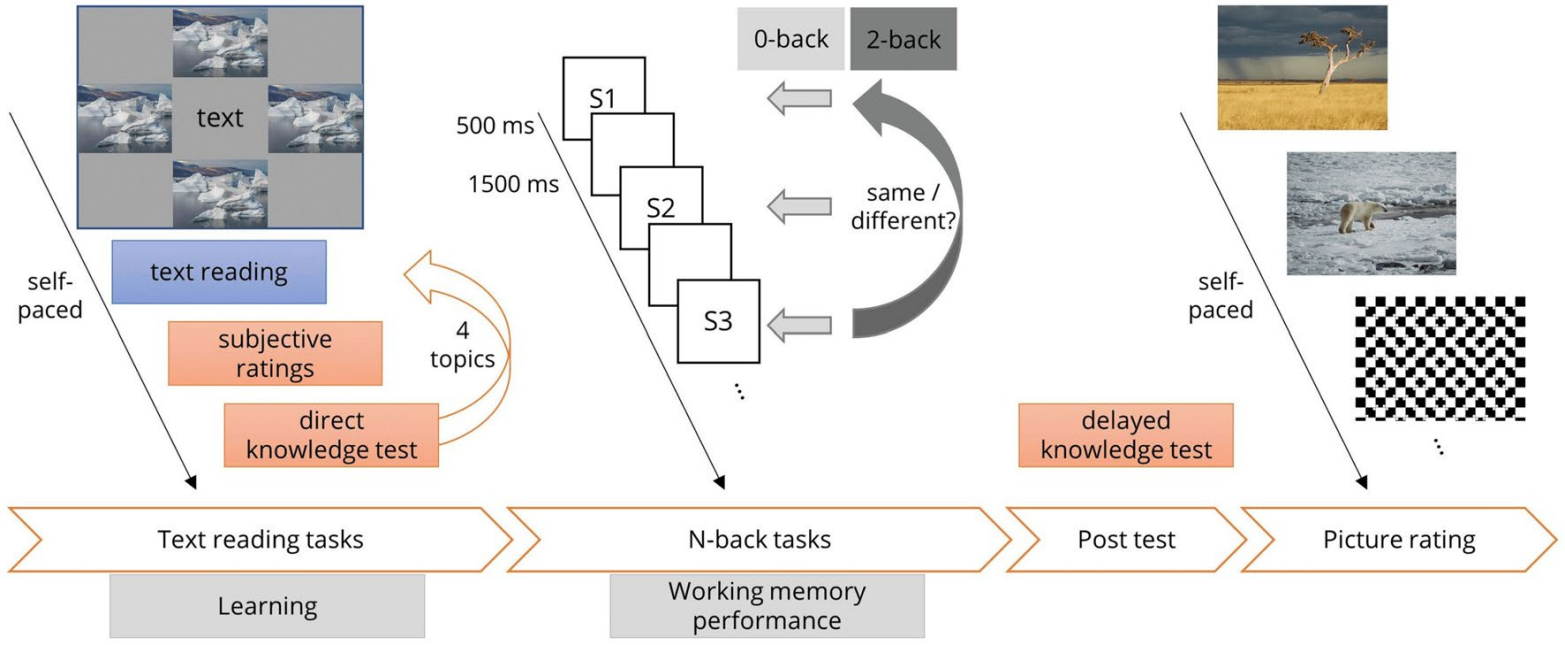
Schematic sequence of all tasks conducted in the current study

All DP had an image size of 400 × 300 px. The DP were presented at four possible positions adjacent to the text area (see Fig. 1, left-hand side for a schematic depiction), either above, below, left, or right, each position used equally often within a text yet in random sequence, one DP per text paragraph. DP at the positions left or right of the text were vertically centered on the screen, about 20 px (0.44° visual angle) distant from the text. Pictures at the positions above or below the text were horizontally centered on the screen, about 50–130 px (1.10–2.87° visual angle), distant from the text, depending on the length of the paragraph. The global luminance of all pictures and the background had been adjusted (GIMP 2.8, www.gimp.org) to match comparable average luminance levels. The average luminance of the photographs, the optical illusions, and the background was 144.73 (*SD* = 16.83), 141.85 (*SD* = 21.11), and 144.00, respectively (RGB luminance values). The DP were collected via a Google picture search (Google.com), stemming from various sources (mostly Wikimedia Commons, commons.wikimedia.org, and Pixabay, pixabay.com).

### N-back working memory tasks

One- or two-syllable single words, centrally presented on the screen, served as stimuli in the n-back tasks. Font type, color, size, and background color used were identical to those of the learning tasks. The words were semantically related to the two global topics of the learning texts; that is, eight words were out of the word field “cold” (i.e., related to the polar topics; German nouns: Schnee, Norden, Arktis, Meereis, Kälte, Frost, Flocken, Kühle [snow, north, Arctic, sea ice, cold, frost, flakes, chilly]), and eight words were out of the word field “heat” (i.e., related to the savannah-topics; German nouns: Tropen, Süden, Wärme, Hitze, Dürre, Sonne, Wüste, Steppe [tropics, south, heat, drought, sun, desert, steppe]). The width of the words on the screen varied between 60 to 90 px depending on word length (1.33–1.99° visual angle). The eight words of the two-word fields were used in separate blocks of the n-back tasks each (i.e., not mixed). Each block consisted of 60 trials, that is, a temporal sequence and repetition of the eight words in pseudo-random order (each word presented for 500 ms followed by a fixation cross of 1500 ms duration; see Fig. 1). One-third of the trials were targets, requiring the participants to press a “target” key; two-thirds were nontargets, requiring the participants to press a “nontarget” key (“l” or “d” on a wired computer keyboard, assignment of keyboard keys, and target decision counterbalanced across participants). The words were presented in blocks of 0-back (low WM load) and 2-back (high WM load) task conditions. In the 0-back blocks, participants had to press the target key whenever a word appeared on the screen that had been defined as a target before the start of the block. In 2-back blocks, participants had to compare every word they saw on the screen with the word they had seen two steps back in the trial sequence and to indicate via keypresses whether the current word was identical (i.e., a target) or different (i.e., a nontarget).

Like the texts in the learning tasks, the n-back stimuli were presented in four different task conditions concerning DP. In the congruent condition (CC), the words of the n-back task and the DP were semantically related (e.g., words of the polar topic and polar DP). In the incongruent condition (IC), the words of the n-back task and the DP were semantically orthogonal (e.g., words of the polar topic and savannah DP). In the distractor condition (DC), the words of the n-back task were presented together with the optical illusions. Finally, in the no-picture condition (NC), the n-back stimuli were presented without pictures. Note that the DP used in the n-back tasks were those of the learning tasks in identical task conditions for each participant. During the n-back blocks of task conditions, CC, IC, and DC 20 DP were presented at four positions adjacent to the n-back stimuli (left, right, above, below), the mean distance between DP and words, depending on word length, 155–170 px (3.43–3.76° visual angle) for DP presented left/right, and 110 px (2.44° visual angle) for DP presented above/below the n-back stimuli. The DP were visible on the screen for four to eight seconds (i.e., one picture at one of the four locations was presented for two to four n-back trials). Each n-back block started with the presentation (5 s) of a centrally positioned fixation cross.

## Procedure

The study consisted of four parts in a direct, fixed sequence. All participants went through all parts and all task conditions (complete within-subjects design). The first part consisted of the preparation of the EEG system. It lasted between 30 and 45 min, during which the participants filled out digitally presented questionnaires (all questionnaires were set up in Sosci Survey, www.soscisurvey.de) for acquiring general subjects’ information (e.g., handedness, prior knowledge, topic interest). After the EEG system had been prepared, the participants performed a simple eye-movement task to record typical patterns of the electro-occulogram (EOG; horizontal and vertical eye movements, blinks) within the EEG. For this, participants had to keep fixated on a fixation cross that appeared several times at the center of the screen and in alternation at four different locations close to the horizontal or vertical borders of the screen. In addition, participants were instructed to blink several times. The EEG data of this task were later used for detecting and cleaning eye-movement artifacts.

The second part of the study consisted of the learning and test phases that were repeated four times; that is, each participant read and was tested for each of the four texts, yet each text was presented in a different task condition. The assignment of texts to task conditions, the sequence of topics, and the sequence of task conditions were counterbalanced across all participants to avoid sequence effects. Notably, each topic occurred in each task condition equally often. In the learning phase, participants read a text on one of the four topics (i.e., arctic fox, polar bear, lion, zebra) in self-paced reading speed (linear navigation between text paragraphs, one paragraph per screen page, i.e., no back-jumps possible). Participants were informed that they would have to answer some knowledge questions after reading. Directly following each text reading, participants’ subjective reading experience concerning performance, effort, and frustration (7-point Likert scales, NASA task load index, NTLX; Hart & Staveland, 1988), mood (7-point Kunin scale), distraction (two items, concentration on the task, tendency of mind-wandering, 9-point Likert scales), interest in the topic (one item, 9-point Likert scale), comprehensibility of the text (two items, 9-point Likert scales), and emotional impression of the text (9-point Likert scale) was assessed. After the subjective ratings, participants performed a knowledge test consisting of eight binary decision questions (true/false) and six multiple-choice questions (each with four response alternatives, one correct). The questions tested mainly factual knowledge but also some transfer knowledge. Yet, a systematic differentiation between factual knowledge and transfer knowledge was beyond the scope of the current study. For statistical analysis of the knowledge test, the average percentage of the correctly answered questions was calculated for each participant. Including breaks, the second part of the study lasted about an hour in total.

The third part of the study consisted of the n-back WM tasks. Participants first conducted a training phase of a 0-back and a 2-back task with detailed instructions and feedback on task performance (the training phase was repeated if task performance in terms of accuracy was below 50% to ensure that participants had correctly understood the task). After that, participants performed the n-back tasks (8 blocks in total, i.e., two n-back load levels, 0-back and 2-back, each in four task conditions) without getting feedback on their performance. The sequence of the n-back blocks was counterbalanced across participants. After each block, participants’ subjective task experience concerning performance, effort, and frustration (NTLX) was assessed. At the end of study part three, participants got the delayed knowledge test consisting of two additional multiple-choice questions per text (i.e., eight questions in total). Including breaks, the third part of the study summed up to about 30 min.

The fourth part of the study consisted of the picture ratings. Each participant judged each DP they encountered during the text reading tasks the fit of the DP to the text, as well as the interestingness, valence, and arousal of the DP (9-point Likert scale). In sum, each participant rated 60 pictures which lasted about 20 min. In total, including breaks, the study lasted about 3 to 3 ½ hours.

## Apparatus

The study was run in an evenly lit, quiet room. Participants sat in a comfortable chair in front of a 24-inch Dell monitor (Dell U 2412 M, 1920 × 1200 px screen resolution, eye-to-monitor distance approx. 70 cm). Stimuli were presented using E-Prime 2.0 (SP2; Psychology Software Tools, Inc., Pittsburgh, PA, USA). At the start of each stimulus (i.e., text paragraph, n-back word), a trigger was sent out to the EEG and the eye-tracking system for later synchronization of the data streams.

A remote eye-tracking system centrally positioned at the lower frame of the monitor recorded participants’ gaze behavior (binocular) at a sampling rate of 250 Hz (SMI RED 250 m, SMI iView Red 4.2.77, SensoMotoric Instruments, Teltow, Germany). A simple chin rest was used to avoid head movements during data recording and to ensure the eyes remained at a fixed distance of about 70 cm from the eye-tracking device. The eye tracker was calibrated before the start of each task using the built-in calibration routines (SMI Experiment Center 3.7.36, 9-point calibration and validation; the calibration was repeated in case of gaze deviations > 1.0° visual angle).

The EEG data were recorded (PyCorder 1.0.9) at 1000 Hz sampling rate (ActiCHamp amplifier, Brainproducts, Ltd., Gilching, Germany) using 32 active electrodes (actiCAP slim electrodes, Brainproducts, Ltd., Gilching, Germany). Impedances were kept below 10 kΩ for the EEG electrodes. 30 electrodes were positioned according to the international 10/20 system (Jasper, 1958) on the scalp (Fp1, Fp2, F7, F3, Fz, F4, F8, FC5, FC1, FC2, FC6, T7, C3, Cz, C4, T8, CP5, CP1, CP2, CP6, P7, P3, Pz, P4, P8, CP3, CP4, POz, O1, O2). Two electrodes were placed on the mastoids, with the right mastoid serving as a reference during recording. The ground electrode was positioned at AFz.

## Data preprocessing and analyses

The EEG and eye-tracking data were analyzed separately for the text reading and the n-back tasks. In addition to the classical stimulus-locked EEG data analysis, the EEG data of the text reading tasks were analyzed fixation-related, based on data epochs time-locked to certain eye fixations. All data analyses share, however, some common preprocessing steps detailed below. Data preprocessing steps were informed by the guidelines of Picton and colleagues and Duncan and colleagues (Duncan et al., 2009; Picton et al., 2000). Data synchronization, preprocessing, and analyses were conducted using customized MATLAB scripts (MATLAB 2018b, MathWorks, Inc., Natick, MA, USA) and the toolbox EEGLAB (v. 14.1.2; Delorme & Makeig, 2004) with the EYE-EEG plugin (v.0.85; Dimigen et al., 2011). The main statistical analyses were conducted within MATLAB (EEGLAB function “statcond”) and using R (v. 4.0.5; R Core Team, 2020), with the packages “ez” (Lawrence, 2016), “emmeans” (Lenth, 2021), and “schoRsch” (Pfister & Janczyk, 2016). For the exploratory correlational analysis, the package “rmcorr” (Bakdash & Marusich, 2017) was used.

First, the single eye-tracking and EEG data recordings were synchronized (EYE-EEG). Event markers (i.e., triggers) at each stimulus onset in both the EEG data and the eye-tracking data, served as synchronization events. In the text reading tasks, the start of each paragraph served as stimulus onset. Overall, the average jitter between the markers in the EEG and the corresponding markers in the eye-tracking data was within ± three data sample (i.e., ± 3 ms). The eye-tracking data were integrated into the EEG data as additional channels (i.e., channels that contain the pupil dilation data of each eye and the raw gaze positions) with the original sampling rate of the eye-tracking data being upsampled (linear interpolation, EYE-EEG plugin) to match the sampling rate of the EEG data.

All single recordings of a participant were then combined in one EEG data file. Based on the algorithm implemented in EYE-EEG for saccade and fixation detection (adaptive velocity-based saccade detection; Engbert & Mergenthaler, 2006), fixations were identified and stored as events along with the EEG data. These identified fixations were later used for the eye-tracking analyses, that is, for calculating the total number of fixations on specific AOIs, the mean fixation durations, and the number of transitions between specific AOIs, as well as for the fixation-related EEG data analysis of the text reading tasks. Blinks in the pupil dilation data were interpolated using an algorithm by Siegle and colleagues (Siegle et al., 2003).

The continuous EEG data were filtered (low-pass 48 Hz, high-pass 0.25 Hz, linear finite impulse response filters). EOG artifacts (eye movements, blinks) were corrected by using independent component analysis (ICA) decompositions (Infomax ICA). Independent components (ICs) visually identified as EOG-ICs were rejected (Delorme et al., 2007; Jung et al., 2000). The visual identification of EOG-ICs was supported by the EEG recording of prototypical eye movements and blinks (i.e., the eye-movement task). For that, the continuous component activity in the time domain was visually inspected and ICs with a specific EOG-related pattern were identified. Furthermore, ICs showing EOG-typical dipoles with frontal locations in the vicinity of the eyes were identified as EOG-ICs. The EEG data then were re-referenced to average reference (the two electrodes positioned at the mastoids being excluded, resulting in 30 symmetrically distributed EEG channels over the scalp in the final data set used for statistical analyses.

Finally, the continuous EEG data were divided into data epochs of 2 s length. Using these epochs, an automatic artifact removal was performed: Epochs that exceeded ± 100 µV were excluded from further analyses (Duncan et al., 2009; Pesonen et al., 2007). No further artifact removal or correction was performed on the EEG data. The n-back tasks were analyzed stimulus-locked only, with the data epochs being time-locked to the onset of each stimulus. Because of the low number of fixations on the DP in the n-back tasks, any meaningful fixation-related EEG data analysis seemed not to be justified.

In contrast, the text reading tasks were analyzed stimulus-locked and fixation-related. In the stimulus-locked analysis, the continuous data recordings during reading were divided into epochs of 2 s length starting time-locked with the presentation of the first paragraph. In the fixation-related analysis, the epochs of 2 s length were specifically time-locked to the onset of the fixations on the DPs. Note that one participant had to be excluded from the fixation-related analysis as no fixations on the DPs in task condition DC were recorded for this participant. In the stimulus-locked analysis of the text reading tasks on average 199.18 (*SD* = 38.71), artifact-free (for definition see above) data epochs of 2 s length per task condition could be used for calculating the EEG frequency band power (see below). In the fixation-related analysis of the text reading tasks on average 18.90 (*SD* = 13.02), artifact-free data epochs of 2 s length per task condition could be used for calculating the EEG frequency band power. In the stimulus-locked analysis of the n-back WM tasks on average 48.51 (*SD* = 8.05), artifact-free data epochs of 2 s length of correctly solved trials (i.e., correct responses) per task condition could be used for calculating the EEG frequency band power.

Using fast Fourier transforms (FFT; 500 ms sliding window, Hanning tapered), the frequency band power values (absolute values) in the frequency range between 2 and 30 Hz (0.125 Hz frequency spacing) were calculated for the data epochs of the different tasks and task conditions. Using the ERD/ERS%-formula (Antonenko et al., 2010; Pfurtscheller & Lopes da Silva, 1999), the percentage of change in the EEG frequency band power in relation to a particular baseline (i.e., the event-related desynchronization and synchronization) was then calculated for the EEG theta (4–6 Hz) and the EEG alpha (8–13 Hz) frequency band. As baselines served the frequency band power of all task conditions of a specific task (i.e., either the text reading tasks or the n-back tasks) averaged together (i.e., whole epoch, global condition baselines; Cohen, 2014). The baselines were calculated individually for each task (i.e., the text reading tasks and the n-back tasks), frequency band (i.e., the theta frequency band and the alpha frequency band), analysis method (i.e., the stimulus-locked analysis and the fixation-related analysis), and participant. Thus, the ERD/ERS%-values indicate a relative increase or decrease in frequency band power for a specific task condition in relation to all task conditions of this specific task averaged together.

Based on literature (e.g., Antonenko et al., 2010; Gevins & Smith, 2000; Palomäki et al., 2012; Scharinger et al., 2015a, 2015b), the typical electrodes for analyzing cognitive load in the EEG were selected for statistical analyses. For the EEG theta frequency band, this was the frontal midline electrode Fz. For the EEG alpha frequency band power, this was the parietal midline electrode Pz. For the text reading tasks one-factorial repeated-measures ANOVAs with the factor *DP* (CC, IC, DC, NC) were conducted separately for each analysis method and frequency band. For the n-back tasks two-factorial repeated-measures ANOVAs with the factor DP (CC, IC, DC, NC) and *load* (0-back, 2-back) were conducted separately for each analysis method and frequency band. Greenhouse–Geisser corrections were performed on the *p*-values where necessary. For post hoc pairwise comparisons (*t*-tests, two-tailed) of significant ANOVA effects, *p*-values were Bonferroni–Holm corrected for multiple comparisons. The level of significance was set at *α* = 0.05 for all analyses, and partial eta square (*η*_p_^2^) is reported as a measure of effect size for the ANOVAs. In addition, for exploratory data analyses and visualizations, to inspect the localization of the theta and alpha ERD/ERS%-changes on the scalp, *topoplots* showing the ERD/ERS%-values at all 30 electrode sites were calculated. Electrodes showing significantly different ERD/ERS%-values (*p* < 0.05) between task conditions (*t*-tests, two-sided, permutation-based statistics, using false discovery rate to correct for multiple comparisons) are marked as red dots in the topoplots.

## Results

The results section is structured as follows. First, I present the subjective ratings of the picture ratings of the DP used. The picture ratings took place at the very end of the study (see Procedure) and are thought to provide an overview of the interestingness, valence, and arousal of the used pictures (i.e., to underline the seductive character of the DP used). Note, as specific motifs of the pictures were needed, pictures of standardized and evaluated picture databases were not suitable for the current study. Second, I present the data of the text reading tasks, starting with the physiological process measures of cognitive load (i.e., pupil dilation, EEG theta, and alpha frequency band power), followed by the eye-tracking measures of attention and integration, and the measures of subjective task experience and objective task performance. After that, the exploratory correlational analysis of the objective and subjective measures of cognitive load is given. Third, using the same structure, I present the data of the n-back WM tasks.

## Picture ratings

Participants rated the fit of the DP and the corresponding text on average as strong for the congruent condition (CC), *M* = 7.58 (*SD* = 1.06) and as weak for the task conditions IC and DC, *M* = 1.36 (*SD* = 0.93) and *M* = 1.32 (*SD* = 0.57), respectively. These outcomes can be seen as a manipulation check indicating the successful manipulation of the text–picture congruency in these task conditions. The average ratings of the interestingness, valence, and arousal of the DP are given in Table 1. The outcomes indicated the potential status of the DP as seductive details. This is because, in line with the definition of seductive details, the pictures were rated as being generally interesting, of neutral-to-positive valence, and, at least to a certain extent, arousing.

**Table 1:** Subjective ratings of the decorative pictures used

Topic	Interestingness	Valence	Arousal	*N*
Arctic fox	6.10 (1.36)	6.59 (1.11)	3.73 (1.36)	15
Polar bear	6.06 (1.27)	6.15 (1.08)	4.19 (1.23)	16
Lion	6.03 (1.69)	6.22 (0.97)	4.25 (0.70)	16
Zebra	5.21 (1.53)	5.96 (1.10)	4.32 (0.78)	16
Distractors	5.15 (1.52)	4.25 (1.29)	6.10 (0.99)	31

Mean rating scores. 9-point Likert scales

*SD* in brackets

## Learning tasks

### Physiological measures of cognitive load

**Fig. 2:**
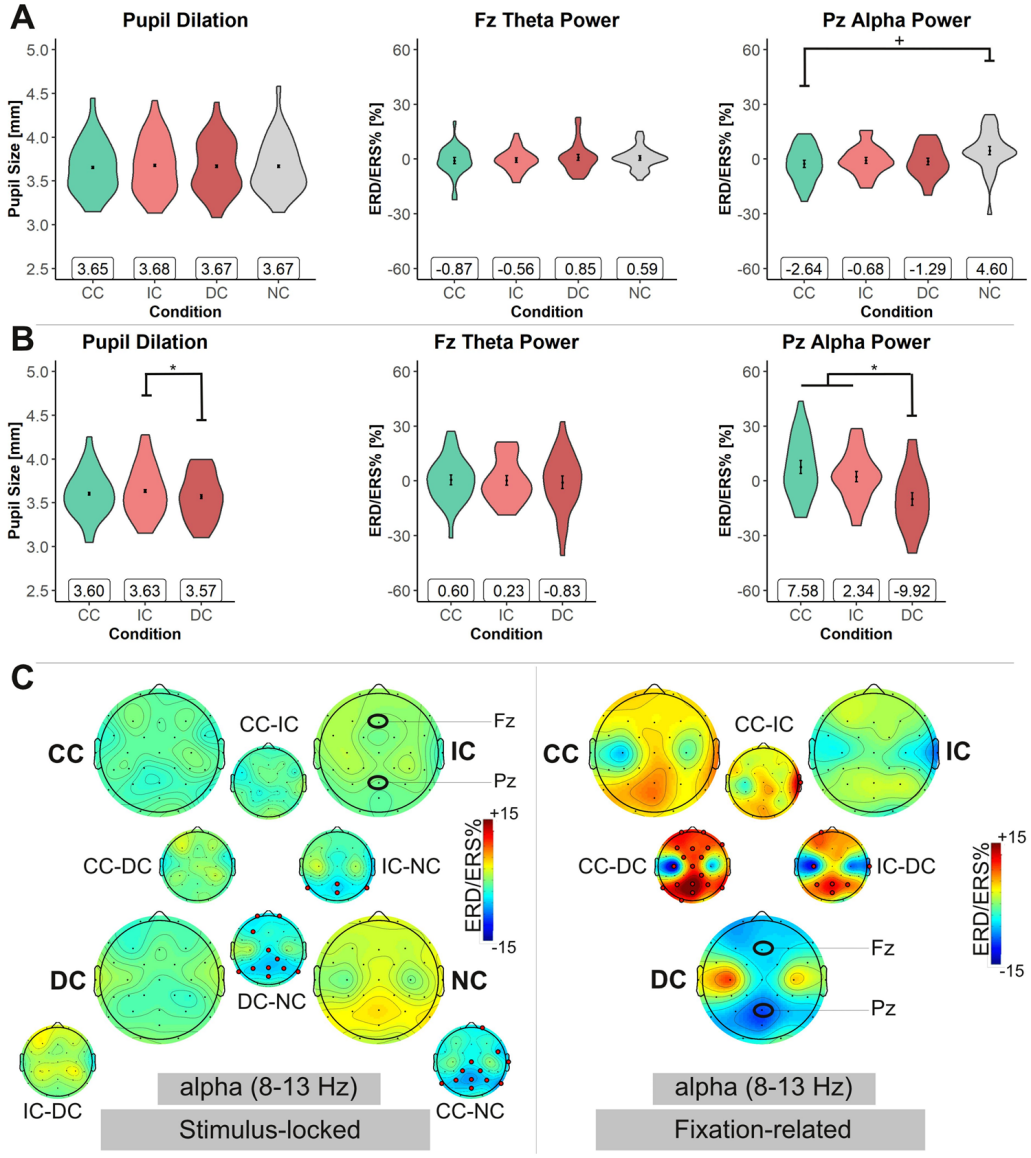
Physiological data of the text reading tasks. *CC* congruent condition of text and pictures, *IC* incongruent condition, *DC* distractor condition, and *NC* text-only. **A** *Violin plots* depicting pupil dilation and the alpha and theta frequency band power (ERD/ERS%) at electrode Pz and Fz, respectively, of the stimulus-locked data analysis (*N* = 32). **B** *Violin plots* of the fixation-related EEG data analysis (*N* = 31). Note. + indicates *p* < 0.06; * indicates *p* < 0.05. Error bars depict the within-subject standard error (Morey, 2008), calculated with the Rmisc package (Hope, 2022). **C** *Topoplots* depicting each electrode’s alpha frequency band power (ERD/ERS%) for the text reading tasks. Left-hand side is the stimulus-locked EEG data analysis (*N* = 32), and right-hand side is the fixation-related EEG data analysis (*N* = 31). Smaller topoplots adjacent to the larger ones indicate the difference between pairs of conditions (i.e., pairwise comparisons). Electrodes marked by red dots show significant differences between task conditions (*t*-tests, two-sided, *p* < 0.05, permutation-based statistics, using false discovery rate to correct for multiple comparisons)

Pupil dilation was analyzed as both stimulus-locked and fixation-related. It was expected that increased cognitive load would result in increased pupil dilation. In the stimulus-locked analysis, there was no significant main effect of *DP*, *F*(3, 93) = 0.64, *p* = 0.592, *η*_p_^2^ = 0.02. The average pupil size was virtually identical for all four task conditions (see Fig. 2, A). In contrast, the fixation-related analysis of pupil dilation revealed a main effect of *DP*, *F*(2, 60) = 3.39, *p* = 0.040, *η*_p_^2^ = 0.10. Pupil size was virtually identically in the CC and IC conditions (*p* > 0.344) but significantly reduced in the DC condition as compared to the IC condition (*p* = 0.035; see Fig. 2B).

The EEG theta ERD/ERS% at frontal electrode Fz was not affected by the experimental manipulations, neither in the stimulus-locked analysis, *F*(2.40, 74.34) = 0.35, *p* = 0.746, *η*_p_^2^ = 0.01, nor in the fixation-related analysis, *F*(2, 60) = 0.06, *p* = 0.939, *η*_p_^2^ < 0.01 (see Fig. 2A and B). In contrast, the alpha frequency band power at parietal electrode Pz showed a significant main effect of *DP* in both analyses, the stimulus-locked, *F*(3, 93) = 2.79, *p* = 0.045, *η*_p_^2^ = 0.08 (Fig. 2A), and the fixation-related analysis, *F*(2, 60) = 7.28, *p* = 0.001, *η*_p_^2^ = 0.20 (Fig. 2B). Post hoc pairwise *t*-tests (Bonferroni–Holm corrected) revealed for the stimulus-locked data the picture-congruent task condition (CC) to result in almost significantly lower ERD/ERS%-values as compared to the text-only task condition (NC), *p* = 0.051. Numerically, the picture-incongruent task conditions (IC, DC) showed ERD/ERS%-values in between the task conditions CC and NC (see Fig. 2A), yet the differences were not statistically significant (all *p* > 0.155). Note that the more liberal correction for multiple comparisons used for the topoplots (permutation-based statistics using false-discovery rate; see Fig. 2C, left-hand side) indicated significantly higher positive ERD/ERS%-values for the NC compared to all three conditions with pictures (i.e., CC, IC, DC).

Interestingly, for the fixation-related data (Fig. 2B), post hoc pairwise comparisons showed a different pattern with the ERD/ERS%-values being significantly lower in the distractor condition (DC) as compared to task conditions CC, *p* = 0.001 and IC, *p* = 0.023, with the latter two being statistically not different, *p* = 0.270. The topoplots (Fig. 2C, right-hand side) indicate that the effects in the alpha frequency band were predominantly at parietal electrodes, as expected. Note, however, that the topoplots of the fixation-related analysis also showed significant differences at frontal electrodes for the pairwise comparison between conditions CC and DC (an outcome not visible for the condition IC-DC). It seems that the alpha frequency band power was more globally affected when comparing task conditions CC and DC.

### Eye-tracking measures of attention and integration

**Fig. 3:**
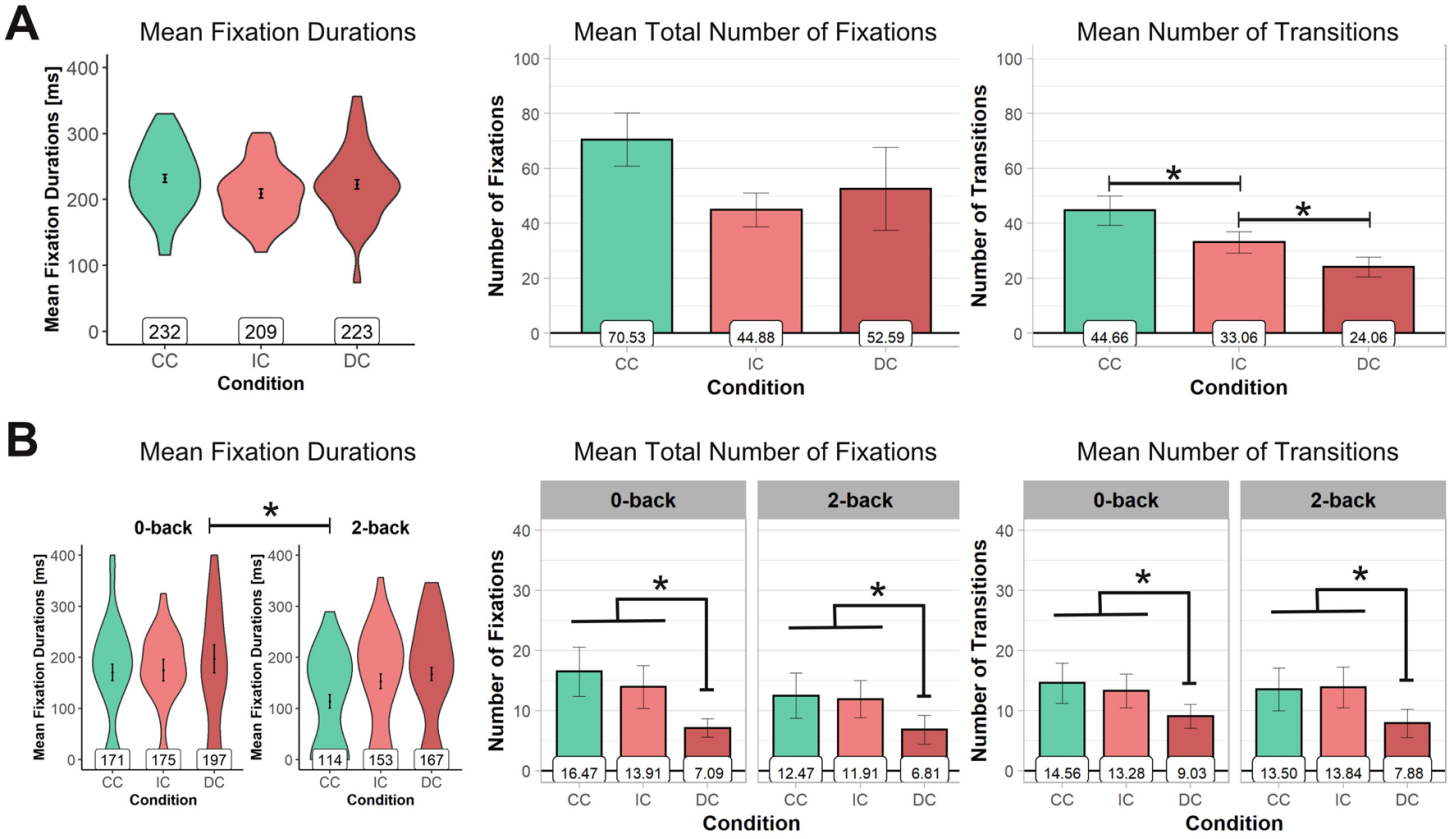
*Violin plots/Bar plots*, showing mean fixation durations for fixations on the pictures, the average total number of fixations on the pictures, and mean total number of transitions (i.e., switches between fixations on the text and the pictures) of the text reading tasks (**A**) and the n-back tasks (**B**). *CC* congruent condition of text and pictures, *IC* incongruent condition, *DC* distractor condition. Error bars =  ± 1 SEM. Note. * indicates significant differences (*p* < 0.05)

Neither the mean fixation durations of the fixations on the DP, *F*(2, 62) = 2.20, *p* = 0.119, *η*_p_^2^ = 0.07, nor the total number of fixations on the DP, *F*(1.41, 43.56) = 3.03, *p* = 0.075, *η*_p_^2^ = 0.09, showed a significant main effect of *DP* (Fig. 3A). Interestingly, there was a significant main effect for the number of transitions between text and DP, *F*(2, 62) = 22.24, *p* < 0.001, *η*_p_^2^ = 0.42. The average total number of transitions (Fig. 3A) was the largest for the picture-congruent task condition (CC: 44.66) as compared to the semantically incongruent text–picture combinations (i.e., task conditions IC, 33.06, and DC, 24.06, all *p* < 0.001) and smallest for the distractor condition (DC, all *p* < 0.005).

### Subjective task experience

Participants judged their learning performance (i.e., learning success) as being equally above the mid of the rating scales for all four task conditions (Table 2, NTLX), without significant differences, that is, no significant main effect of *DP*, *F*(3, 93) = 1.89, *p* = 0.137, *η*_p_^2^ = 0.06. However, depending on task conditions, participants had to invest more effort to achieve their performance, *F*(2.29, 71.11) = 4.66, *p* = 0.009, *η*_p_^2^ = 0.13. Numerically, the semantically conflicting picture conditions (IC, DC) resulted in higher effort ratings as compared to the congruent picture condition (CC) and the text-only condition (NC). Post hoc pairwise comparisons indicated statistically significant (or almost significant) differences between conditions DC and CC, *p* = 0.001, DC and NC, *p* = 0.054, and IC and CC, *p* = 0.058 (all other differences, *p* > 0.206).

**Table 2:** Learning tasks: performance measures and subjective ratings

Cond	Task performance data			Subjective ratings	
	RT	Test I	Test II	Mood	Distraction*
CC	6.55 (1.29)	80.60 (15.30)	82.81 (30.08)	5.31 (0.82)	3.70 (1.43)
IC	6.38 (1.30)	75.78 (17.26)	82.81 (27.27)	5.16 (0.88)	4.50 (1.90)
DC	6.60 (1.46)	81.84 (14.63)	81.25 (27.68)	5.06 (1.13)	4.67 (1.87)
NC	6.50 (1.37)	78.65 (13.45)	89.06 (21.00)	5.22 (0.94)	3.50 (1.31)

Cond	Ratings: NTLX			Ratings: task materials		
	Perform	Effort*	Frustration	Interest	Compr	Emotion
CC	5.16 (1.19)	3.88 (1.50)	2.13 (1.41)	6.75 (1.48)	7.72 (0.96)	3.72 (2.20)
IC	4.84 (1.11)	4.59 (1.41)	2.16 (1.11)	6.19 (1.99)	7.63 (1.07)	3.19 (1.65)
DC	4.78 (1.18)	4.81 (1.35)	2.50 (1.50)	6.75 (1.16)	7.19 (1.20)	3.16 (1.94)
NC	5.19 (1.09)	4.06 (1.16)	1.88 (1.13)	6.72 (1.61)	7.34 (1.12)	3.31 (1.73)

Mean reading times (RT) in minutes

*Test I* direct knowledge test and *Test II* delayed knowledge test, each with % correct responses

SD in brackets

NTLX 7-point Likert scales, subjects’ mood 7-point Kunin scales, and all other subjective ratings (subjects’ distraction, interestingness, comprehensibility, and emotionality of the task materials) 9-point Likert scales

*CC* congruent condition of text and pictures, *IC* incongruent condition, *DC* distractor condition, *NC* text-only

Asterisk (*) indicates dependent variables with significant main effect (p < 0.05) of the repeated-measures ANOVA

Although, numerically, the text-only condition (NC) seems to be perceived as slightly less frustrating as compared to the reading conditions with DP (see Table 2, NTLX), this effect was statistically not significant, *F*(2.25, 69.86) = 2.47, *p* = 0.085, *η*_p_^2^ = 0.07. Also, the general mood of the participants was not affected differently by the task conditions, *F*(3, 93) = 0.85, *p* = 0.472, *η*_p_^2^ = 0.03.

There was a main effect of *DP* for distraction, *F*(3, 93) = 7.25, *p* < 0.001, *η*_p_^2^ = 0.19, indicating that participants felt more distracted when semantically incongruent pictures were presented alongside the text (i.e., conditions IC and DC) as compared to congruent pictures or text-only (i.e., conditions CC and NC, all *p* < 0.031), with virtually no difference between the pairings IC and DC or CC and NC (all *p* = 1.00). The perceived interestingness, *F*(3, 93) = 1.74, *p* = 0.163, *η*_p_^2^ = 0.05, comprehensibility, *F*(3, 93) = 2.60, *p* = 0.056, *η*_p_^2^ = 0.08, and emotionality, *F*(3, 93) = 1.66, *p* = 0.181, *η*_p_^2^ = 0.05, of the task materials were virtually not affected by the task conditions.

### Learning task performance

Participants reading times (Table 2) did not differ significantly between the four task conditions, *F*(3, 93) = 0.60, *p* = 0.619, *η*_p_^2^ = 0.02. Also, the learning outcomes were not significantly affected by the different task conditions, neither for the knowledge tests directly following each text reading, *F*(3, 93) = 1.57, *p* = 0.201, *η*_p_^2^ = 0.05, nor for the delayed knowledge test, *F*(3, 93) = 0.55, *p* = 0.647, *η*_p_^2^ = 0.02 (Table 2).

### Learning tasks: correlational analysis

Exploratorily, the correlations (repeated-measures correlation coefficient, Bakdash & Marusich, 2017; see Table 3) between the physiological proxies of cognitive load (i.e., pupil dilation, EEG theta and alpha frequency band power; stimulus-locked only) and subjectively perceived effort (i.e., the effort ratings) were analyzed. To correct for multiple comparisons, Bonferroni corrections of the *p*-values were used. As can be seen in Table 3, with respect to the relation between subjective measures of effort and objective measures of cognitive load, there was a significant negative correlation between subjectively perceived effort and pupil dilation. Obviously, participants with decreased pupil dilation reported increased effort. This outcome was opposite to the expected pattern of increased pupil dilation for increased effort based on literature (e.g., Mathôt, 2018; van der Wel & van Steenbergen, 2018). The negative correlation between the EEG alpha frequency band power and pupil dilation was in line with expectations (i.e., alpha power decreases, pupil dilation increases for increased cognitive load). Yet, surprisingly, there was also significant positive correlation between EEG theta frequency band power and EEG alpha frequency band power (instead of the expected negative correlation because of the detrimental reaction of these measures for the cognitive load reported in the literature). Also, surprising was the significant negative correlation between the EEG theta power and pupil dilation (see Discussion section for addressing these rather unexpected patterns of results).

**Table 3:** Learning tasks: repeated-measures correlations between subjectively perceived effort and the physiological proxies of cognitive load

	1	CI	2	CI	3	CI
1. Effort						
2. Pupil dilation	−0.29*	[−0.47, −0.10]				
3. Theta power	0.26	[0.06, 0.43]	−0.44***	[−0.59, −0.26]		
4. Alpha power	0.20	[0.00, 0.38]	−0.30*	[−0.47, −0.10]	0.40***	[0.21, 0.55]

Repeated-measures correlation coefficients (Bakdash & Marusich, 2017), stimulus-locked data only

*Indicates *p* < 0.05; **, *p* < 0.01; ***, and *p* < 0.001

Bonferroni-corrected *p*-values to correct for multiple comparisons

*CI* 95% confidence intervals of the repeated-measures correlation coefficient

*n* = 32, all four experimental conditions included

## N-back working memory tasks

### Physiological measures of cognitive load

For the n-back tasks, pupil dilation and EEG theta and alpha frequency band power data were only analyzed stimulus-locked. This is because, in the n-back tasks, the DP had too few fixations to allow any meaningful fixation-related analysis.

**Fig. 4:**
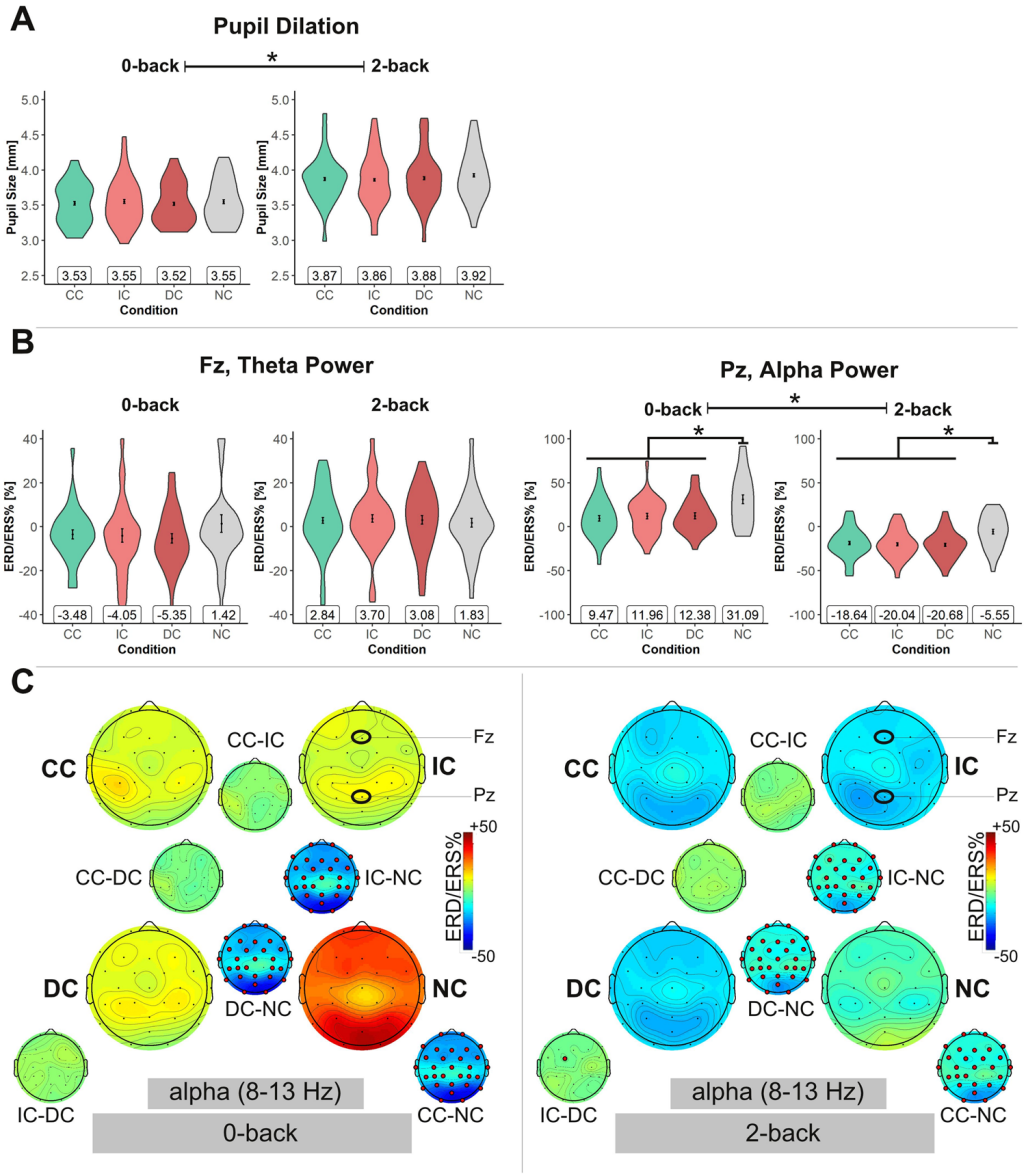
Physiological data of the n-back tasks. *CC* congruent condition of text and pictures, *IC* incongruent condition, *DC* distractor condition, *NC* text-only. **A** *Violin plots* depicting pupil dilation data. **B** *Violin plots* showing the EEG alpha and theta frequency band power (ERD/ERS%) at electrode Pz and Fz, respectively, of the stimulus-locked data analysis (*N* = 32 Note. * indicates *p* < 0.05. Error bars depict the within-subject standard error (Morey, 2008), calculated with the Rmisc package (Hope, 2022). **C** *Topoplots* depicting the alpha frequency band power (ERD/ERS%) of each electrode for the n-back tasks. Left-hand side: 0-back tasks, and right-hand side: 2-back tasks. Smaller topoplots adjacent to the larger ones indicate the difference between pairs of conditions (i.e., pairwise comparisons). Electrodes marked by red dots show significant differences between task conditions (t-tests, two-sided, *p* < 0.05, permutation-based statistics, using false discovery rate to correct for multiple comparisons)

For the pupil dilation, repeated-measures ANOVAs revealed a main effect of *load*, *F*(1, 31) = 84.63, *p* < 0.001, *η*_p_^2^ = 0.73. As expected, under high WM load (i.e., high n-back levels; see Fig. 4 A), the size of the pupils was larger compared to low WM load (all *p* < 0.001). Pupil dilation was not affected by the DP, no main effect of *DP*, *F*(3, 93) = 0.68, *p* = 0.569, *η*_p_^2^ = 0.02, and no interaction, *F*(3, 93) = 1.50, *p* = 0.220, *η*_p_^2^ = 0.05.

In the theta frequency band at electrode Fz (Fig. 4 B, left-hand side), there were no statistically significant effects, neither for *load*, *F*(1, 31) = 1.64, *p* = 0.210, *η*_p_^2^ = 0.05 nor *DP*, *F*(2.04, 63.38) = 0.39, *p* = 0.683, *η*_p_^2^ = 0.01, and also no interaction between the two factors, *F*(2.31, 71.64) = 1.38, *p* = 0.257, *η*_p_^2^ = 0.04.

The EEG alpha frequency band power at electrode Pz (Fig. 4 B, right-hand side), however, showed a main effect of *load* as expected, *F*(1, 31) = 43.22, *p* < 0.001, *η*_p_^2^ = 0.58, with the ERD/ERS%-values being lower (i.e., more negative) in the 2-back as compared to the 0-back task conditions. In addition, the EEG alpha frequency band ERD/ERS% showed a main effect of *DP*, *F*(2.08, 64.33) = 12.16, *p* < 0.001, *η*_p_^2^ = 0.28. All task conditions with DPs resulted in significantly lower ERD/ERS%-values as compared to the task condition without DPs (all *p* < 0.001), with the three DP conditions being statistically virtually identical (all *p*s = 1.00). The interaction was not significant, *F*(3, 93) = 1.01, *p* = 0.394, *η*_p_^2^ = 0.03. As for the text reading tasks, the topoplots (Fig. 4 C) indicate that the effects in the alpha frequency band were widely distributed across the scalp with a maximum at parietal electrodes, as expected.

### Eye-tracking measures of attention and integration

For the mean fixation durations, repeated-measures ANOVAs only revealed a main effect of *load*, *F*(1, 31) = 4.68, *p* = 0.038, *η*_p_^2^ = 0.13, but no effect of *DP*, *F*(1.58, 48.85) = 2.06, *p* = 0.147, *η*_p_^2^ = 0.06, and no interaction, *F*(2, 62) = 0.46, *p* = 0.634, *η*_p_^2^ = 0.01. As can be seen in Fig. 3 (B), mean fixation durations were shorter in the 2-back load conditions as compared to the 0-back load conditions. Repeated-measures ANOVAs revealed a main effect for the factor *DP* for the number of fixations on the picture AOIs, *F*(2, 62) = 7.82, *p* = 0.001, *η*_p_^2^ = 0.20, yet no effect for *load*, *F*(1, 31) = 0.64, *p* = 0.429, *η*_p_^2^ = 0.02, and no interaction, *F*(2, 62) = 0.78, *p* = 0.463, *η*_p_^2^ = 0.02. For the distractor pictures (i.e., task condition DC), there were significantly fewer fixations on the picture AOIs as compared to both the task condition CC and the task condition IC (all *p* < 0.009), with the latter two being statistically not different (*p* = 0.439). Finally, there was a main effect of *DP* for the number of transitions, *F*(2, 62) = 7.71, *p* = 0.001, *η*_p_^2^ = 0.20, with the number of transitions between DP and n-back stimuli being significantly lower in the distractor task condition (DC), as compared to both the CC (*p* = 0.002) and the IC (*p* = 0.004), with the latter two being statistically not different (*p* = 0.767). No other effects were observed for the number of transitions between n-back stimuli and DP, that is, no main effect of *load*, *F*(1, 31) = 0.07, *p* = 0.790, *η*_p_^2^ < 0.01, and no interaction, *F*(2, 62) = 0.26, *p* = 0.773, *η*_p_^2^ = 0.01.

### Subjective task experience

As revealed by 2-factorial repeated-measures ANOVAs, subjective ratings of perceived task performance, *F*(1, 31) = 89.14, *p* < 0.001, *η*_p_^2^ = 0.74, effort, *F*(1, 31) = 67.47, *p* < 0.001, *η*_p_^2^ = 0.69, and frustration, *F*(1, 31) = 38.27, *p* < 0.001, *η*_p_^2^ = 0.55, were significantly affected by the n-back levels. Increased n-back levels resulted in decreased subjective performance judgments and increased ratings of effort and frustration (see Table 3). The factor *DP* did not show significant effects for any of the dependent variables, task performance, *F*(3, 93) = 0.79, *p* = 0.501, *η*_p_^2^ = 0.02; effort, *F*(3, 93) = 1.74, *p* = 0.165, *η*_p_^2^ = 0.05; frustration, *F*(3, 93) = 1.08, *p* = 0.361, *η*_p_^2^ = 0.03). Also, the interaction between *load* and *DP* was not significant for any of the subjective ratings; perceived task performance, *F*(3, 93) = 0.14, *p* = 0.934, *η*_p_^2^ < 0.01; effort, *F*(2.40, 74.32) = 0.35, *p* = 0.744, *η*_p_^2^ = 0.01; frustration, *F*(2.18, 67.65) = 1.54, *p* = 0.219, *η*_p_^2^ = 0.05).

### N-back task performance

For accuracy (see Table 4), the 2-factorial repeated-measures ANOVA only revealed a significant main effect of *load*, *F*(1, 31) = 54.77, *p* < 0.001, *η*_p_^2^ = 0.64, with accuracy being overall reduced in the 2-back as compared to the 0-back level (*p* < 0.001). Neither the factor *DP* showed a significant effect, *F*(2.29, 70.85) = 2.36, *p* = 0.094, *η*_p_^2^ = 0.07, nor the interaction between *load* and *DP* was significant, *F*(2.30, 71.32) = 0.59, *p* = 0.582, *η*_p_^2^ = 0.02.

**Table 4:** N-back tasks: performance measures and subjective ratings

Cond	Accuracy [%]*		Reaction times [ms]*	
	0-back	2-back	0-back	2-back
CC	95.26 (3.26)	78.18 (12.78)	494 (75)	742 (183)
IC	95.26 (6.60)	79.80 (11.97)	493 (94)	761 (175)
DC	94.42 (11.17)	79.74 (12.48)	482 (80)	754 (182)
NC	96.60 (2.81)	82.42 (13.39)	467 (69)	754 (192)

Cond	NTLX: perform.*		NTLX: effort*		NTLX: frustration*	
	0-back	2-back	0-back	2-back	0-back	2-back
CC	5.28 (1.46)	3.28 (1.37)	4.38 (1.45)	5.94 (1.05)	2.13 (1.31)	3.69 (1.84)
IC	5.34 (1.58)	3.13 (1.39)	4.50 (1.59)	6.06 (1.05)	2.63 (1.90)	3.72 (1.99)
DC	5.34 (1.41)	3.22 (1.41)	4.53 (1.32)	6.06 (0.88)	2.41 (1.58)	3.97 (2.04)
NC	5.56 (1.34)	3.38 (1.36)	4.19 (1.47)	5.91 (0.89)	2.13 (1.26)	3.94 (1.88)

Mean values, SD in brackets

Bold digits indicate significant effects. NTLX 7-point Likert scales

*CC* congruent condition of text and pictures, *IC* incongruent condition, *DC* distractor condition, *NC* text-only

Asterisk (*) indicates dependent variables with significant main effect (*p* < 0.05) of the repeated-measures ANOVA

For reaction times (RT, see Table 4), there was also a significant main effect of *load*, *F*(1, 31) = 108.04, *p* < 0.001, *η*_p_^2^ = 0.78, with reaction times being overall increased in the 2-back as compared to the 0-back level (see Table 3). Again, neither the factor *DP*, *F*(2.23, 69.06) = 1.06, *p* = 0.359, *η*_p_^2^ = 0.03, nor the interaction between *load* and *DP* was significant, *F*(3, 93) = 1.68, *p* = 0.1.77, *η*_p_^2^ = 0.05.

### N-back tasks: correlational analysis

Exploratorily, the correlations (repeated-measures correlation coefficient, Bakdash & Marusich, 2017; see Table 5) between the physiological proxies of cognitive load (i.e., pupil dilation, EEG theta and alpha frequency band power; stimulus-locked only), subjectively perceived effort, and behavioral task performance (i.e., reaction times, accuracies) were analyzed. To correct for multiple comparisons, Bonferroni corrections of the *p*-values were used. Except for the EEG theta power that did not show any significant correlation with none of the other dependent variables, the subjectively perceived effort, behavioral performance, and the physiological proxies of cognitive load correlated significantly with one another in an expected direction. Effort correlated negatively with accuracy and positively with reaction times (i.e., lower task performance was mirrored by increased effort ratings). Effort also correlated positively with pupil dilation and negatively with the EEG alpha frequency band power, in line with the expectation of increased pupil dilation and decreased alpha frequency band power for increased cognitive load (and hence effort). Behavioral performance, pupil dilation, and the EEG alpha frequency band power also correlated in the expected directions (see Table 5) indicating that decreased task performance (i.e., lower accuracies, increased reaction times) are reflected by increased pupil dilation and decreased EEG alpha frequency band power.

**Table 5:** N-back working memory tasks: Repeated-measures correlations between subjectively perceived effort, behavioral performance, and the physiological proxies of cognitive load

	1	CI	2	CI	3	CI	4	CI	5	CI
1. Effort										
2. Accuracy	−0.50***	[−0.59, −0.40]								
3. Reaction times	0.67***	[0.59, 0.74]	−0.58***	[−0.66, −0.49]						
4. Pupil dilation	0.47***	[0.36, 0.56]	−0.47***	[−0.56, −0.36]	0.69***	[0.62, 0.76]				
5. Theta power	0.07	[−0.06, 0.20]	−0.07	[−0.20, 0.06]	0.10	[−0.04, 0.22]	0.09	[−0.04, 0.22]		
6. Alpha power	−0.46***	[−0.56, −0.35]	0.43***	[0.31, 0.53]	−0.56***	[−0.64, −0.46]	−0.57***	[−0.65, −0.47]	−0.01	[−0.14, 0.12]

Repeated-measures correlation coefficients (Bakdash & Marusich, 2017)

***Indicates *p* < 0.001

Bonferroni-corrected *p*-values to correct for multiple comparisons

*CI* 95% confidence intervals of the repeated-measures correlation coefficient

*n* = 32, all eight (i.e., two n-back load levels, four DP conditions) experimental conditions included

## Discussion

The current study examined the effects of DP on cognitive load during multimedia learning and WM performance measured by the EEG alpha and theta frequency band power and pupil dilation. Eye-tracking served as a proxy of the visual attention on the screen (i.e., number of fixations, fixation duration, number of transitions) and additionally allowed to analyze the EEG and pupil dilation data during reading fixation-related, that is, when the DP were fixated at.

The main results were that DP had no significant effects on the behavioral performance measures, neither for the learning tasks (i.e., text reading times or learning outcomes) nor for the n-back WM tasks (i.e., reaction times or accuracies). Second, despite the no effects on the behavioral performance measures, the EEG alpha frequency band power indicated increased cognitive load for DP present in both tasks. Yet, in the text reading tasks, the EEG and the pupil dilation results differed depending on whether the data was analyzed stimulus-locked or fixation-related. Third, neither pupil dilation nor the EEG theta frequency band power showed a clear effect for DP. Fourth, the eye-tracking data indicated that especially the congruent DP during text reading showed higher numbers of transitions between text and picture than incongruent or distractor DP. Finally, subjective ratings did not indicate a significant effect of DP on emotional–motivational factors like participants’ mood during learning or interest in the learning materials. However, in the text reading tasks, the subjectively perceived effort was increased for the distractor DP. In the following, I will discuss these outcomes in more detail.

## Effects of DP on physiological measures of cognitive load

DP had significant effects on the cognitive load as assessed by the EEG alpha frequency band power at parietal electrodes (i.e., electrode Pz) in both the text reading and the n-back WM tasks. For the text reading tasks, two results are especially noteworthy. First, according to the stimulus-locked EEG data analysis, congruent DP numerically resulted in the largest relative decrease in EEG alpha frequency band power (i.e., highest cognitive load) compared to the text-only condition. This result was somewhat unexpected, as the cognitive load was expected to be most pronounced for incongruent DP (i.e., task conditions IC and DC). This is because, incongruent DP might hamper the construction of a congruent mental model as textual and pictorial information did not match (DC) or even were conflicting (IC). Yet, albeit speculative at his point, the decreased EEG alpha power for congruent DP might indicate additional processes of integrating the pictorial information with the textual integration. These processes might also increase the overall cognitive load as indicated by the EEG alpha frequency band power. This reasoning is corroborated by the eye-tracking data showing the largest number of transitions in condition CC. The number of transitions has been reported as a proxy for integration processes of textual and pictorial information (Mason, Pluchino, et al., 2013; Mason, Tornatora, et al., 2013).

Second, according to the fixation-related EEG data analysis, the decrease in EEG alpha power was more pronounced for the incongruent DP (IC) and especially the distractor DP (DC); that is, the cognitive load seemed to be highest for incongruent distractor DP as expected. Interestingly, the viewing pattern (i.e., the low number of transitions in the eye-tracking data) of the condition DC compared to CC and IC indicates that participants barely tried to integrate distractor DP into a coherent mental model with the text. Nevertheless, according to the fixation-related EEG data analysis with the EEG alpha frequency band power being most reduced in condition DC compared to IC and CC, the cognitive load seems to be most pronounced in this condition. Potentially (but quite speculative at this point), the increased cognitive load for DC is rather related to processes of inhibition or cognitive control than to participants’ difficulties to integrate pictorial and textual information. The subjective ratings on distraction, numerically being highest for the condition DC might be seen corroborating this interpretation. Future studies might try disentangling processes of hampered text–picture integration from processes of increased distraction (and hence demanded inhibitory processes of cognitive control) in more detail.

The different outcomes of the fixation-related and the stimulus-locked EEG analysis concerning cognitive load and text–picture congruency might be explained by the different selections of data epochs for the EEG data analysis. For the fixation-related EEG data analysis, only data epochs of two seconds in length, each time-locked to the fixations on the DP, were analyzed. Thus, this analysis is timely more local, potentially tapping more closely into the cognitive load directly related to viewing the DPs. Locally only, the cognitive load might be increased for incongruent DP compared to congruent DP. This is because, participants might be irritated by the semantically unexpected, nonmatching DP and might try to inhibit further processing. On a sentence level, incongruent pictures were reported to result in increased cognitive load as measured by the EEG alpha frequency band power (Scharinger et al., 2020). This explanation might specifically be valid for the distractor DP (DC). The distractor DP depicted abstract optical illusions that were visually interesting (Stevanov et al., 2012a, 2012b) and quite arousing (see Table 1). Participants’ cognitive load might have been increased by the optical illusions rather in the short run when the participants were trying to make sense out of the illusions. However, in the long run, the optical illusions (and also the incongruent DPs of task condition IC) might be identified as task-unrelated, thus not increasing the overall cognitive load more than the other DPs (i.e., in the stimulus-locked analysis).

Potentially, the different number of epochs per condition that defined the data used for calculating the EEG frequency band power and running the statistical analyses might be considered as an alternative explanation for the different outcomes of the stimulus-locked as compared to the fixation-related analysis regarding the EEG alpha power. The number of epochs used in the fixation-related analysis was only about one-tenth of the number of data epochs used for the stimulus-locked analysis (see Method section). This was because the DP overall only got few fixations. The actual number of fixation-related data epochs even was lower than the average total number of fixations as only artifact-free epochs were used in the final data analysis (see data preprocessing). Yet, on average about 20 epochs per task condition seem to be acceptable for an EEG frequency band power analysis (e.g., Cohen, 2014). A lower number of trials typically increase the noise in the data (Picton et al., 2000); that is, potential effects might be masked. Therefore, it seems rather unplausible that the different outcomes in the fixation-related analysis compared to the stimulus-locked analyses are attributable to the different number of data epochs only.

In contrast to the fixation-related analysis, the stimulus-locked analysis might provide a more global picture of the cognitive load, that is, the overall processing demands of the entire task. The global cognitive load might be increased more for congruent DP than incongruent DP, as congruent DP might have been considered task-relevant by the participants (although they were not). Consequently, congruent DP (as compared to incongruent or distractor DP) might have been processed more thoroughly and integrated into participants’ mental model of the text (i.e., the congruent DP leading to a multimedia effect; Mayer, 2014b). The eye-tracking data of the current study corroborate this interpretation. Congruent DP were (numerically) more often fixated at as compared to incongruent distractor DP. Most importantly, the number of transitions between text and picture was the largest for the task conditions with congruent DP. In eye-tracking studies, the number of transitions has typically been interpreted to reflect the integration of pictorial and textual information (Mason, Pluchino, et al., 2013; Mason, Tornatora, et al., 2013; Schüler, 2017; Schüler et al., 2019). Such integration processes for congruent DP might occur during the entire text reading and thus might result in the increased cognitive load observed in the global, stimulus-locked EEG data analysis. The subjective ratings corroborate the interpretation that coherent DP are actively integrated into participants' mental model of the text. The overall comprehensibility of the learning materials was scored numerically largest for congruent DP present, indicating that the congruent DP were subjectively judged helpful for learning (although learning outcomes were not affected by DP). Future studies are necessary to investigate these interpretations further. Yet, the results of the current study underline the value of simultaneously analyzing different process measures such as EEG and eye-tracking in one study. The different process measures can mutually supplement each other, thus providing a more thorough, coherent interpretation of the multimedia effect.

Interestingly, the fixation-related analysis of the pupil dilation data in the text reading tasks that was suggested by one anonymous reviewer also showed different outcomes compared to the stimulus-locked analysis. The stimulus-locked analysis of pupil dilation data did not show any significant difference in pupil size for the task conditions. In contrast, in the fixation-related analysis, the pupil dilation was decreased for the distractor DP compared to the incongruent DP. This pattern of results is not easily interpretable, as it seems to contradict the fixation-related EEG alpha power outcomes. Potentially, as the distractor DP were grayscale only and depicting rather simple, geometrical forms this result might reflect luminance differences on a local level rather than different cognitive processing. Potentially, because of the free-viewing situation, the pupil dilation data might also been confounded by the pupil foreshortening error (see introductory section; e.g., Hayes & Petrov, 2016; Mahanama et al., 2022; Petersch & Dierkes, 2022) Clearly, future studies are necessary to address these observations in more depth.

The topoplots indicate that the decreased alpha power in the DP conditions compared to the text-only condition (stimulus-locked analysis) and the decreased alpha power for the distractor DP compared to the CC and IC in the fixation-related analysis were mainly at parietal electrodes (i.e., electrodes in the vicinity of Pz), as expected. Furthermore, the EEG alpha effects seem to be rather symmetrically distributed at the parietal electrodes (i.e., no clear hemispheric difference is visible). The EEG alpha effects are commonly widely distributed over the scalp with maxima at parietal electrodes (Krause, 2003). Thus, the topoplots underline observing a typical alpha frequency band power effect for the cognitive load manipulations. Importantly, focusing on single electrodes as done in the main statistical analyses seems therefore justified not only by literature (e.g., Antonenko et al., 2010; Gevins & Smith, 2000) but also by the current topoplots (showing the parietal location of the alpha power effects). This outcome is noteworthy, as it shows that future studies might record the EEG from only few, a priori selected electrodes, nevertheless being able to assess differences in cognitive load by means of the EEG alpha frequency band power. Using few (or even single) electrodes for data recording would clearly increase the overall ecological validity of using EEG to study multimedia learning. This is because it would dramatically reduce the time needed for the EEG preparation. Therefore, such an EEG recording would be as minimally intrusive as possible.

For the n-back tasks, EEG alpha frequency band power was relatively decreased in all task conditions with DP present as compared to the task conditions without DP. Thus, the overall outcome can be seen in line with the text reading tasks. Unfortunately, due to the overall too-low numbers of fixations on the picture AOIs, a fixation-related EEG data analysis could not be done for the n-back tasks. Thus, it remains elusive whether the distractor DP would also have resulted in the most pronounced cognitive load in a fixation-related analysis. Nevertheless, adding the n-back WM tasks to the learning tasks does provide some interesting additional insights and helps judging the sensitivity and validity of the measures used to assess cognitive load.

As expected, the n-back WM load manipulation led to a substantial increase of cognitive load in the 2-back compared to the 0-back load condition. This was reflected by the relative decrease in the EEG alpha frequency band power but also in an increase in pupil dilation. Together with the observation that the numeric effects on the EEG alpha power are far less prominent for the manipulations of DP as compared to the n-back load-level manipulation, these outcomes might indicate that EEG alpha frequency band power is more sensitive in assessing changes in cognitive load as pupil dilation. It seems that if the changes in cognitive load are relatively large (like for the n-back load manipulation), both measures show an effect. This outcome underlines, first, the use of adding the EEG to multimedia studies and, second, the use of adding an additional, established WM task as a testbed for the physiological measures when interested in assessing cognitive load.

The topoplots for the n-back tasks indicated that the effects of the EEG alpha frequency band power were largely distributed across nearly all electrodes for both the DP and then-back load manipulation. Nevertheless, parietal electrodes numerically showed a maximal effect, as expected. Taken together, the topoplots indicate that changes in EEG frequency band power are often rather globally distributed. This fits with the observation that cortical activity in the EEG alpha frequency range reflects rather globally cognitive processing (Krause, 2003; see also section Limitations).

The EEG theta frequency band power did not show any effects in none of the tasks or for none of the experimental manipulations. This might be surprising as, at least for the n-back load manipulation, a relative increase in theta frequency band power has been reported for increasing n-back levels (e.g., Gevins & Smith, 2000; Scharinger et al., 2015a, 2015b). Important to note that the n-back tasks of these studies typically used extremely minimalistic stimuli (e.g., single digits or letters). Thus, it might be possible that potential effects in the theta frequency band were attenuated because of the more complex stimuli used in the current study (i.e., words and pictures). Yet, irrespective of the concrete reasons for the absent EEG theta effects, these outcomes underline the value of additionally having a well-established neurophysiological task like the n-back task when interested in EEG measures of cognitive load. If only the EEG data of the text reading tasks had been available, one might have questioned the validity of the EEG alpha power effects as these effects were not mirrored by the theta frequency band power. With the additional results of the n-back tasks showing a highly comparable pattern of results concerning both, the alpha frequency band power and the theta frequency band power, the validity of the alpha frequency band power is confirmed, and the EEG alpha frequency band power has been identified as the most sensitive measure for the cognitive load.

Pupil dilation did not show an effect for DP in the stimulus-locked analyses of both tasks. For the n-back load levels, however, pupil dilation showed the expected effect (i.e., increased pupil dilation for increased n-back load). Taken together, this could indicate that pupil dilation is a less sensitive measure of cognitive load compared to the EEG alpha frequency band power. Alternatively, although the luminance of the task materials was controlled for on a global level, the pupil dilation data might have been confounded by luminance differences on a local level (i.e., depending on which parts of a DP were fixated at; e.g., Mathôt et al., 2015). Furthermore, the eye movements might have additionally influenced the pupil dilation data (i.e., the pupil foreshortening error; Carter & Luke, 2020; Hayes & Petrov, 2016; Mahanama et al., 2022; Petersch & Dierkes, 2022). Interestingly, however, despite such potential and inevitable confounds in multimedia task materials (i.e., combinations of text and pictures), the pupil dilation indicated the increased cognitive load for the n-back load levels. Thus, pupil dilation might be less sensitive in the context of complex multimedia task materials to indicate cognitive load, but still usable. Pupil dilation seems to be a suitable measure even in multimedia task materials as long as the cognitive load manipulation is strong enough and as long as potential confounding factors are considered as potential alternative explanations for the effects observed.

Distractor DP (i.e., condition DC) in the fixation-related analysis resulted in the most pronounced decrease of EEG alpha power compared to CC and IC. Furthermore, the topoplots indicated that the differences in EEG alpha power between conditions DC and CC were rather globally distributed over the scalp (i.e., showing maxima not only at parietal electrodes but also at frontal electrodes). Thus, in the current study, distractor DP seem to affect cognitive load most strongly Albeit speculative, an explanation for that could be the numerically higher arousal levels of the DC-pictures (see Table 1). Furthermore, as the distractor DP depicted optical illusions, participants might have invested (and might have had to invest) more effort in understanding these DP compared to the realistic photographs (CC, IC). This interpretation is corroborated by the subjective effort ratings indicating increased effort for DC compared to CC and (at least numerically) IC.

## Effects of DP on eye-tracking measures of attention and integration

The eye-tracking data revealed that the DP overall attracted some but only a little attention in both tasks. Especially in the n-back tasks, the average total number of fixations on the DP was low. This outcome is in line with other eye-tracking studies on DP in text reading (e.g., Lenzner et al., 2013), indicating that participants might be well aware of which parts of the task materials are relevant and should be primarily processed for adequate task performance.

Interestingly, one of the most informative measures regarding effect sizes and discriminability of the three DP conditions was the number of transitions between text and picture. This measure differentiated between congruent, incongruent, and distractor DP in the text reading tasks and the realistic DPs (i.e., the photographs) and distractor DPs in the n-back tasks, respectively. In both tasks, the number of transitions was lowest for the distractor DP, indicating that these pictures were identified as not relevant to the primary task. Hence, participants might not have tried to relate the textual information and the pictorial information semantically or, in the case of the learning tasks, to integrate these pictures into a coherent mental model of the text (Mason, Pluchino, et al., 2013; Mason, Tornatora, et al., 2013; Schüler, 2017; Schüler et al., 2015). In the n-back tasks, the higher number of transitions for photographs (irrespective of congruency) might indicate that participants try to relate the n-back stimuli and the DP semantically. This interpretation might also fit for the learning tasks, with the addition that the overall largest number of transitions for congruent DP might indicate a multimedia effect, that is, the integration of the textual and pictorial information and building a coherent mental model in this task condition.

## Effects of DP on subjective task experience

From an emotional design hypothesis (Brom et al., 2018; Heidig et al., 2015), DP would have been expected to be beneficial for learning and task performance. This is because DP were thought to increase the overall liking of and interest in the task materials and because DP may induce a positive mood or increase participants’ general motivation. The current study showed that the DP had no beneficial effects on learning or WM task performance. None of the subjective ratings related to emotional–motivational processes (e.g., frustration, mood, perceived emotionality of the learning task materials, or interest therein) did show any significant effects for DP. Thus, although the DP per se were rated as interesting and of rather positive valence by the participants, the DP did not affect the overall subjective impression of the task materials. One potential reason why the DP did not increase the subjectively perceived interestingness of the learning task materials might be that the texts (i.e., the learning contents) were already rated quite interesting (i.e., scores in the upper half of 9-point Likert scales; see Table 2). In addition, participants stated a genuine interest in learning some facts about the learning topics in the pre-task ratings (see section Participants). Therefore, as participants’ interest in learning was already high and the texts were rated as interesting per se, potentially the DP would have had been more spectacular to increase participants’ interest further. In future studies, learning materials might be chosen that are genuinely far less interesting to enhance the potential emotional–motivational effects of DP.

Interestingly, in the text reading tasks, the subjectively perceived effort was numerically increased for the incongruent DP conditions (IC, DC) compared to the text-only condition. However, despite the significant main effect, the post hoc pairwise comparisons only showed marginally significant differences. In the n-back tasks, subjective effort ratings clearly showed the differences between the n-back load levels but no effect for the DP manipulation. Taken together, subjective effort ratings seem to be suitable to detect larger differences in cognitive load (and task difficulty as induced by the n-back load levels), but less sensitive to subtle changes in cognitive load as those potentially induced by the DP. This can be seen as an additional argument for the use of the EEG alpha frequency band power as a sensitive proxy of cognitive load.

## Effects of DP on task performance

Observing no effects on behavioral performance measures in both, the reading and the n-back tasks is especially noteworthy, as the EEG alpha frequency band power indicated increased cognitive load for DP in both tasks. Furthermore, DP impacted subjects’ attentional focus, as indicated by the eye-tracking data. Yet, observing no effects for seductive details like DP on task performance in multimedia learning tasks (especially concerning learning outcomes) is in line with several previous studies (e.g., Chang & Choi, 2014; Lenzner et al., 2013; Lindner, 2020; Park & Lim, 2007). Several potential boundary conditions for the seductive details effect of pictures and text have been discussed (Alexander, 2019; Rey, 2012; Sundararajan & Adesope, 2020). Seductive details like DP might especially have detrimental effects on learning when the WM capacity of the learners is low (Sanchez & Wiley, 2006), when a fixed time limit exists for learning (Rey, 2012), or when the learners consider the seductive details as relevant for learning (Eitel et al., 2019). Participants in the current study were university students only. Albeit somewhat speculative, this might have been a subject sample that generally has larger WM capacities. This is because, WM capacity and academic achievements have been shown to be closely linked (Alloway & Alloway, 2010; Diamond, 2013; Hall et al., 2015; St Clair-Thompson & Gathercole, 2006; Swanson & Alloway, 2012). In addition, the learning task was self-paced (i.e., without a strict time limit). Thus, participants might have been able to compensate for the additional information of the DP to be processed (albeit reading times or reaction times did not differ significantly between DP conditions). More importantly, because of the relatively large number of DP used in the current study (i.e., 20 DP per text, one per paragraph) and the repeated-measures design, the participants might have quickly learned that the DP were not task-relevant, which consequently might have prevented a potential seductive details effect (Rop et al., 2016, 2017, 2018).

DP, like those used in the current study, have not been used before spatially aside from n-back stimuli. Generally, pictures spatially aside n-back stimuli are seldom used. One of the rare examples is a study by Ladouceur and colleagues (Ladouceur et al., 2009) that used pictures of fearful faces alongside n-back stimuli and observed detrimental effects on task performance, but mainly for participants high in trait anxiety. The DP used in the current study might have resulted in no effects on WM task performance as they were not task-relevant, of neutral-to-positive valence, and of average arousal levels only (Ribeiro et al., 2019; Schweizer et al., 2019). In line with an attentional shielding hypothesis, participants might have primarily focused on the n-back task and the task-relevant stimuli therein (SanMiguel et al., 2008; Scheiter et al., 2014; Sörqvist & Marsh, 2015); thus, detrimental effects might have been prevented.

### Correlational analyses

The exploratory correlational analyses that had been suggested by one anonymous reviewer using repeated-measures correlational coefficients (Bakdash & Marusich, 2017) revealed for the n-back tasks highly plausible correlational patterns for the subjective effort ratings, behavioral performance measures, and the physiological data, except for the EEG theta frequency band power. The EEG theta frequency band power did not show any significant correlation with none of the other dependent variables of interest in the n-back tasks. As the EEG theta frequency band power also showed no effects for the load manipulations in the n-back tasks, this outcome might indicate that this measure showing inconclusive results might have been confounded by eye-movement artifacts, despite the correction for eye-movement artifacts applied. This might be especially because of the free-viewing situation and the use of pictures aside from the stimuli resulting in increased eye movements as compared to a classical n-back paradigm with only stimuli presented centrally on the screen. As effects of the EEG theta frequency band power related to cognitive load are typically observed at frontal electrodes (i.e., electrode Fz; Gevins & Smith, 2000; Pesonen et al., 2007; Scharinger et al., 2015a, 2015b) that are in close vicinity of the eyes, this explanation might be plausible, yet should be addressed in more detail in future studies.

For the text reading tasks, the correlational patterns of subjectively perceived effort and the physiological proxies of cognitive load overall was rather unexpected and inconclusive. The observed negative correlation between effort and pupil dilation was as unexpected as the negative correlation between theta power and pupil dilation and the positive correlation between theta power and alpha power. While the latter two might be due to the general problem of getting clean EEG theta frequency band power effects in the current study (see discussion above), the former might be an additional hint that especially in the text reading tasks the pupil dilation data were not free of confounding factors like luminance confounds (due to the different DP) or measurement artifacts like the pupil foreshortening error (Carter & Luke, 2020; Hayes & Petrov, 2016; Mahanama et al., 2022; Petersch & Dierkes, 2022). Yet, this interpretation is rather speculative at this point and might be addressed in more depth in future studies. Interestingly, however, there are some studies that also report inconclusive or absent correlations between pupil dilation and the EEG frequency band power (Miles et al., 2017; Scharinger et al., 2015a, 2015b). In line with these authors, one might speculate that the unexpected correlational outcomes might indicate pupil dilation and the EEG frequency band power to reflect different aspects of cognitive load. Alternatively, interindividual differences in the reactivity of the different physiological measures might play a role (Doppelmayr et al., 1998; Neubauer et al., 2006). Clearly, future research might focus more on a correlational account (that was not the primary focus of the current research) to clarify these partly unexpected correlational outcomes. Important to note that for a meaningful correlational analysis, the number of participants in the current study might be considered as rather low. In fact, the a priori power analysis was only conducted for the originally planned main analyses (i.e., the repeated-measures ANOVAs). The correlational analyses were added upon the request of one anonymous reviewer. Thus, the rather inconclusive outcomes of the correlational analysis for the text reading tasks might be partly due to a low *N*.

## Limitations of the current study

The current study showed the value of the EEG alpha frequency band power as a quite reliable and sensitive proxy of cognitive load in both text reading and WM tasks. The additional analysis of a neurophysiological well-established, highly controlled WM task helped in judging the sensitivity and data quality of the process measures, especially the EEG. However, some limitations of the current study have to be addressed, first from the perspective of multimedia learning research. Most obviously, the ecological validity of the current learning tasks might be critically questioned. Typically for EEG studies, the present study had a complete within-subjects, repeated-measures task design. That is, each participant got four times a learning task (albeit each with a different text and in a different task condition concerning DP). Furthermore, 20 DPs (one per paragraph) were used per text. Thus, learning in the current study might differ from traditional school learning with textbooks in class. Yet, it resembles informal learning, like doing a Web search and reading parts of several websites, each decorated with several DPs. Such a scenario with multiple documents and DP in digital media might be similar to the learning scenario of the current study.

Nevertheless, the repetitive use of several, yet different, DP might have weakened the overall effects of the DPs. Participants might have gotten used to the learning tasks and the general DP manipulations over the course of the experiment. Over time, people can learn to ignore irrelevant, distractive information. This has been shown for multimedia learning (Rop et al., 2016, 2018) and might be a reason for the so-called banner blindness in website reading (Hsieh et al., 2012; Resnick & Albert, 2014). However, to avoid habituation and effects like banner blindness for the DP, the DP in the current study pseudo-randomly occurred at four different positions on the screen. Although an inevitable weakening of the effects of DP due to the task design cannot be entirely ruled out as a potential reason for the lack of effects on the performance measures (e.g., the learning outcomes), the EEG alpha frequency band power did show effects for the DP. This underlines the sensitivity of this measure to assess cognitive load in the current task design.

Important to note that concerning learning outcomes, the current study only focused on rather short-term learning success, with the knowledge test directly after the learning task (test 1) or about half an hour later after the n-back task (test 2). Potentially, adding congruent pictures to text might result in positive effects on memory and learning specifically in the long run (i.e., when tested after weeks or months) and not so much in the short run. This is because congruent pictures might enrich the mental model and might strengthen memory traces due to dual coding (Carney & Levin, 2002; Clark & Paivio, 1991). Future studies might address this interesting research question on the effects of DP (i.e., seductive details) on learning and memory when the time to test is taken into account (i.e., by analyzing additional delayed knowledge tests that take place some weeks or months after the initial learning phase).

The learning outcomes in the current study were overall rather high. Therefore, a potential ceiling effect cannot be fully ruled out as an alternative explanation for not observing an effect of DP on learning outcomes. In line with this reasoning, one might alternatively postulate that instead of the EEG alpha power being a quite sensitive proxy of cognitive load, the current learning items were a quite insensitive measure of load-related effects. Clearly, future studies should use more difficult learning content and test items to avoid this potential problem. Yet, with the pupil dilation as an additional proxy of cognitive load showing no effects for DP, the conclusion that the EEG alpha band power turned out as a highly sensitive (and reliable) proxy of cognitive load in the current study still seems to be plausible.

Note that the average outcomes of the second knowledge test (i.e., the “delayed” knowledge test after the n-back tasks) were numerically higher than the outcomes of the direct knowledge test. As the specific items for both tests were fixed, that is, bound to the corresponding test, these differences in overall mean learning outcomes between the tests could be simply due to differences in the difficulty of the test items. Future studies might avoid such confounds by randomly assigning the test items to the different tests.

From a methodological point of view, two important aspects have to be stressed. First, the EEG alpha and theta frequency band power and pupil dilation are not pure measures of cognitive load but can be regarded as proxies for cognitive load. This is because, albeit all of these measures have been reported to reflect changes in participants’ cognitive load (see introductory section; e.g., Gevins & Smith, 2000; Kahneman & Beatty, 1966; Laeng et al., 2012; Palomäki et al., 2012; Pesonen et al., 2007; Scharinger et al., 2020), none of these measures exclusively reacts to changes in cognitive load only. For example, the eye pupil also dilates for emotional stimuli as compared to neutral ones (Bradley et al., 2008; Partala & Surakka, 2003), thus being a rather global measure of arousal. Likewise, the EEG alpha frequency band power seems to not only reflect cognitive load but also effort, attention, or semantic processing (e.g., Klimesch, 1999). Cognitive load in the current study was understood in a rather broad sense, namely as the sum of cognitive processes actively required for task performance (i.e., without differentiating between intrinsic or extraneous load, as in the cognitive load theory, Sweller et al., 1998, or between perceptual load and memory load like Lavie, 2005). In that sense, the physiological measures may be seen to actually reflect cognitive load. Albeit interesting, any further differentiation was beyond the scope of the current study.

Second, albeit the fixation-related analysis is a very interesting methodology specifically for analyzing (realistic) multimedia task materials, it has one major task-inherent drawback when addressing DP and the seductive details effect. To validly analyze EEG data, one needs to have a certain minimal number of data epochs (i.e., trials) for the analysis (e.g., Picton et al., 2000). If DP were only seldom fixated at (which was specifically the case for the n-back tasks in the current study), a fixation-related analysis is not possible. Therefore, if it is not fully clear beforehand how much visual attention the AOIs will get, it might be a valuable advice for future studies to always ensure an alternative data analysis method for the EEG (like the stimulus-locked analysis in the current study).

Finally, one might ask about the role of interindividual differences affecting the current results. While such an analysis of the data was beyond the scope of the current research, it is plausible to assume that interindividual differences might in principle affect the data from two perspectives. First is the perspective on the task materials (i.e., DP conditions) with pictorial seductive details. Whether subjects are distracted by seductive details may be by large depending on their cognitive control abilities, which are known to vary substantially between individuals (e.g., Filippatou & Pumfrey, 1996; Heitz & Engle, 2007; Unsworth & Robison, 2017; Willows, 1978). Second is the perspective on the physiological measures. The EEG frequency band power has been reported to show some interindividual differences in the magnitude of the reactivity to external or internal stimulation or the individual positions of the exact frequency band limits (e.g., Doppelmayr et al., 1998; Neubauer et al., 2006). The reactivity of the pupil dilation might also be individually different and may for example depend on the subjects’ age (e.g., Beatty & Lucero-Wagoner, 2000; Van Gerven et al., 2004). However, in the current study because of the complete within-subjects design, these potentially individual differences should have been controlled for by large. Nevertheless, I cannot fully exclude the possibility that such interindividual differences might have introduced some additional noise in the data, attenuating some of the expected effects. Potentially, especially the exploratory correlational analyses might have been affected by such interindividual differences, as for such analyses the *N* was rather low. Future studies with larger *N* might have a closer look at the correlational perspective and interindividual differences for this kind of more complex task materials with text and pictures.

## Conclusion

The current study underlined the validity of the EEG alpha frequency band power as a sensitive and valid measure of cognitive load in both, multimedia text reading and n-back WM tasks. Task-irrelevant decorative pictures increased the cognitive load, yet they did not affect task performance. Fixation-related EEG data analysis could provide additional insights into the locally increased cognitive load when incongruent DPs were fixated at. In sum, the study showed the potential of using EEG and eye-tracking to analyze multimedia task materials. This methodology might be used in future studies on the design of digital interfaces by allowing them to objectively assess the cognitive load during task conduction.

## Data Availability

The data that support the findings of this study are available from the corresponding author upon request.
